# Molecular networking reveals indole diterpenoids from the marine-derived fungus *Penicillium* sp. N4-3

**DOI:** 10.1007/s42995-024-00274-6

**Published:** 2025-04-07

**Authors:** Min Chen, Bao-Cong Hao, Xia-Hao Zhu, Li-Kui Zhang, Yao-Yao Zheng, Xiao-Jian Zhou, Till F. Schäberle, Li Shen, Chang-Yun Wang, Yang Liu

**Affiliations:** 1 Marine Science & Technology Institute, College of Environmental Science & Engineering, Yangzhou University, 196#, Huayang West Street, Yangzhou, 225127 China; 2 Key Laboratory of Marine Drugs, The Ministry of Education of China, Institute of Evolution & Marine Biodiversity, School of Medicine and Pharmacy, Ocean University of China, Qingdao, 266003 China; 3 Laboratory for Marine Drugs and Bioproducts, Qingdao National Laboratory for Marine Science and Technology, Qingdao, 266237 China; 4 Institute for Insect Biotechnology, Justus-Liebig-University Giessen, 35392 Giessen, Germany; 5 Fraunhofer Institute for Molecular Biology and Applied Ecology (IME), Branch for Bioresources, 35392 Giessen, Germany; 6 German Center for Infection Research (DZIF), Partner Site Giessen-Marburg-Langen, 35392 Giessen, Germany; 7 Jiangsu Key Laboratory of Integrated Traditional Chinese and Western Medicine for Prevention and Treatment of Senile Diseases, Medical College, Yangzhou University, Yangzhou, 225001 China

**Keywords:** Molecular networking, Marine-derived fungus, *Penicillium*, Indole diterpenoid, Shearinine, MS/MS

## Abstract

MS/MS-based molecular networking is an effective strategy to rapidly dereplicate known compounds and to guide the discovery process for new and novel natural products. In the present study, the chemical diversity of indole diterpenoids from the marine-derived fungus *Penicillium* sp. N4-3 was investigated using molecular networking techniques. Guided by this information, targeted isolation resulted in two new indole diterpenoids shearinines R and S (**1**, **2**) and an oxidative artifact shearinine T (**3**), together with the verification of two known analogs (**4**, **5**). Furthermore, five indole diterpenoids (**6**−**10**), including three putatively new ones, shearinines U−W (**6**, **9**, **10**), were predicted from the molecular ion cluster by the combination of GNPS molecular networking and manual analysis of MS/MS fragmentation clusters. Shearinines T (**3**) and W (**10**) are characterized by an oxidative cleavage of the C-2–C-18 double bond. Feature fragment ions of these shearinines revealed two type of dominant ions related to the indole moiety and the breaking of C-9 side chain or Ring I. Compound** 1** showed antibacterial activities against a panel of pathogenic bacteria with IC_50_ values ranging from 6.34 to 47.96 μg/mL and inhibited the growth of the human hepatic (HepG2) and gastric (SGC-7901) cancer cells lines with IC_50_ values of 6.27 and 19.16 μg/mL, respectively.

**Supplementary Information:**

The online version contains supplementary material available at 10.1007/s42995-024-00274-6.

## Introduction

Marine natural products as a unique source of new pharmaceutical molecules have sparked an upsurge in research around the world. Plentiful drug leads were discovered from marine natural products in the past few decades (Ancheeva et al. 2018; Haefner 2003; Han et al. 2022). Especially, secondary metabolites from marine-derived fungi have received considerable attention due to their intriguing structural diversity and potent biological activities (Newman and Cragg 2020; Patridge et al. 2016), with a steady and continued growth in the number of new molecules (Carroll et al. 2021 and 2022). In parallel with these advances, however, re- “discovery” of known compounds has become a serious problem in traditional separation approaches, making the discovery of new bioactive metabolites extremely challenging. Therefore, there is an urgent need to apply effective strategies early in the workflow to avoid re-separation of known structures. This provides guidance and improves efficiency in the discovery of molecules showing biological activity and/or chemical novelty.

In the last decade, MS/MS-based molecular networking has emerged as a promising technique for de-replication in natural products discovery workflows (Yang et al. 2013). Because structurally similar molecules share similar MS/MS fragmentation patterns, molecular networking enables clustering and visualization of related molecules (Olivon et al. 2017). Using ever-growing databases of MS/MS results, e.g., Global Natural Products Social Molecular Networking (GNPS), enables automatic comparisons between data. If one node in the network (i.e., fragmentation data of a specific ion) matches with stored data, it can be dereplicated using small amounts of sample. Furthermore, it is expected that other nodes, which cluster with the dereplicated one, can be rapidly resolved based on mass differences and characteristic fragment ions. To date, molecular networking has been widely used by the community for dereplication and discovery of new bioactive substances of therapeutic interest (Li et al. 2020; Nothias et al. 2018; Zang et al. 2020).

In recent years, our laboratory has been devoted to the study of marine natural products, and a plethora of bioactive secondary metabolites with novel structures have been discovered from marine invertebrates and marine-derived microorganisms collected from the South China Sea (Chen et al. 2017 and 2019, Liu et al. 2019, Zheng et al. 2019). Nevertheless, our team also faced the challenge of dereplicating known compounds. Therefore, a molecular networking approach has been used in our laboratory to guide the isolation of new bioactive natural products, especially for the discovery of peptides. This resulted in isolation of novel cycloheptapeptides, asperversiamides A–C (Hou et al. 2019a), asperheptatides A−D (Chao et al. 2021) and asperpyrroindotide A (Han et al. 2023), cyclohexadepsipeptides, chrysogeamides A−G (Hou et al. 2019b), and prenylated indole alkaloids, notoamide X (Hao et al. 2024), from marine-derived microorganisms. Some of which were found to show significant inhibitory activity against *Mycobacterium marinum* (Hou et al. 2019a) and *M. tuberculosis* (Chao et al. 2021) or promote angiogenesis in zebrafish (Hou et al. 2019b).

As a part of our ongoing research on bioactive secondary metabolites from marine-derived microorganisms, the fungus *Penicillium* sp. N4-3 attracted our attention, due to the fact that the EtOAc extract of the fungal culture showed significant antibacterial activity against a panel of pathogenic bacteria and the remarkable dark blue spots in thin layer chromatography analysis (Supplementary Fig. S28). Analysis of curated molecular networks generated from the extracts of this fungus allowed identification of the “molecular family” of indole diterpenoids. Then targeted isolation was conducted to obtain four indole diterpenoids (**1**, **2**, **4**, **5**) together with an oxidative artifact (**3**), and five further analogs (**6**−**10**) present in the molecular ion cluster were identified based on manual analysis of MS/MS fragmentation clusters. In this paper, we report the isolation, structure elucidation, and biological activity of these indole diterpenoids. The plausible MS/MS feature fragmentation patterns of these indole diterpenoids are also discussed.

## Material and methods

### General experimental procedures

Optical rotations were measured on an Anton Paar MCP300 automatic polarimeter. UV spectra were obtained on a Beckman DU 640 spectrophotometer. ECD spectra were recorded on a JASCO J-810 Circular Dichroism Spectrometer. IR spectra were recorded on a Cary 610/670 spectrometer using KBr pellets. NMR spectra were acquired using an AVANCE 600 or a Bruker AV-400 NMR spectrometer with TMS as an internal standard. HRESIMS spectra were obtained from a maXis spectrometer. Semi-preparative HPLC was performed on a HITACHI system using a semi-preparative C_18_ column (Kromasil, 5 μm, 10 × 250 mm) coupled with a 2400 UV detector. HPLC–MS/MS was performed on a Waters 2695 HPLC instrument coupled with an ESI-Ion Trap Bruker amazon SL mass spectrometer with a C_18_ column (YMC-Park, 5 μm, 4.6 mm × 250 mm). Silica gel (Qingdao Mar. Chem. Ind. Co. Ltd.; 200–300 mesh), octadecylsilyl silica gel (YMC Co. Ltd.; S–50 μm), and Sephadex LH-20 (Amersham Biosciences Corp.) were used for column chromatography (CC). Pre-coated silica gel plates (Yan Tai Zi Fu Chemical Group Co.; G60, F-254) were used for thin layer chromatography (TLC).

### Fungal material

The fungus *Penicillium* sp. N4-3 was isolated from the mangrove rhizosphere soil, which was collected from the Dongzhaigang mangrove natural reserve in Hainan Island in December 2017. The strain was deposited at the Marine Science & Technology Institute, College of Environmental Science & Engineering, Yangzhou University, Yangzhou, PR China. The fungus was identified according to its morphological traits and a molecular protocol by amplification and sequencing of the DNA of the ITS region of the rRNA gene. The fungus was identified as a *Penicillium* sp. whose 531 base pair ITS sequence had 99.06% sequence identity to that of *Penicillium simplicissimum* isolate 79 (MH137657.1). The sequence data have been submitted to GenBank with accession number OP835837.1.

### Fermentation and extraction

The fungal strain was cultivated on potato glucose broth (PDB) medium (20 g of glucose, 30 g of sea salt in 1 L of potato infusion; 1 L Erlenmeyer flasks each containing 400 mL of culture broth) at 25 ℃ without shaking for 1 month. The culture (30 L) was filtered to separate the broth from the mycelia. Then the broth was extracted three times with an equal volume of EtOAc, and the mycelia were extracted three times with MeOH. The organic extracts were combined and concentrated under vacuum to afford a total extract (broth extract and mycelia extract, 57 g).

### LC–MS/MS and molecular networking analysis

The fungal extract was first pretreated by silica gel CC using gradient elution of EtOAc–petroleum ether and MeOH–EtOAc to remove the small polar oil components and the large polar sugars and salts, and then two fractions (Fr.1 and Fr.2) were taken that showed characteristic dark blue spots in TLC analysis. Fr.1 and Fr.2 (1 mg/mL, 8 *μ*L) were analyzed by LC–MS with a gradient program of MeOH–H_2_O (0−20 min 10−90%, 20−25 min 90−100%, 25 − 30 min 100%; 0.8 mL/min; MS scan 100−2000 Da) and then with an automated full-dependent MS/MS scan.

Molecular networking was performed using the GNPS data analysis workflow using the spectral clustering algorithm. All MS/MS data were converted to mzXML format files using MSConvert software. Data analysis included the following parameters: cosine threshold set at 0.66 value, precursor mass tolerance of 1.0 Da, fragment mass tolerance of 0.3 Da, minimum number of matched peaks per spectral alignment of 4, consensus spectra for cosine score higher than 0.66, parent mass tolerance of 1.0 Da, minimum percentage of matched peaks in a spectral alignment of 40%, using global natural products social molecular networking platform (GNPS, http://gnps.ucsd.edu). The spectral networks were imported into Cytoscape 3.8.0 and visualized using the force-directed layout.

### Isolation

Fr.2 was subjected to Sephadex LH-20 CC eluted with a mixture of CH_2_Cl_2_–MeOH (v/v, 1:1) and then was further purified by an ODS column eluted with 85% MeOH–H_2_O to give the mixture of **1** and **4**, which were further separated by Sephadex LH-20 CC eluted with a mixture of CH_2_Cl_2_–MeOH (v/v, 1:1) to obtain **1** (46.0 mg) and **4** (12.3 mg). Fr.1 was isolated on silica gel CC using gradient elution of EtOAc–petroleum ether to give Fr.1–1 and Fr.1–2. Fr.1–1 was further purified by HPLC (85% MeOH–H_2_O) to give **5** (*t*_R_ = 40 min, 29.3 mg). Fr.1–2 was subjected to Sephadex LH-20 CC eluted with a mixture of CH_2_Cl_2_–MeOH (v/v, 1:1) and then was further purified by an ODS column eluted with 90% MeOH–H_2_O to give **2** (5.4 mg), which was further oxidized to afford **3**.

### Shearinine R (1)

Brown oil. [*α*] ^20^_D_ − 215 (*c* = 0.10, MeOH). UV (MeOH) *λ*_max_ (log *ε*) 367 (0.5), 285 (2.3), 259 (1.6), 204 (1.3) nm. ECD (0.25 mmol/L, MeOH) *λ*_max_ (Δ*ε*) 373 (− 24.6), 324 (+ 26.5), 281 (− 13.3), 251 (+ 18.3), 224 (+ 10.9), 205 (− 12.5) nm. IR (KBr) *ν*_max_ 3329, 2975, 2934, 1658, 1456, 1375, 1258, 1167, 1132, 1010, 868, 534, 458. ^1^H NMR (CDCl_3_, 400 MHz) and ^13^C NMR (CDCl_3_, 100 MHz), see Table 1; HRESIMS *m/z* 604.3042 [M + Na]^+^ (calcd. for C_37_H_43_NNaO_5_^+^, 604.3033).

**Table 1:** ^1^H and ^13^C NMR spectroscopic data for **1 − 3** (CDCl_3_, TMS, *δ* × 10^-6^, *J* in Hz)

Position	**1**^*a*^		**2**^*b*^		**3**^*b*^	
	*δ*_C_, type	*δ*_H_, mult. (*J* in Hz)	*δ*_C_, type	*δ*_H_, mult. (*J* in Hz)	*δ*_C_, type	*δ*_H_, mult. (*J* in Hz)
1		7.81 (brd, 6.4)		7.76 (s)		
2	151.4, C		148.2, C		175.3, C	
3	50.8, C		48.7, C		55.5, C	
4	43.2, C		42.8, C		43.7, C	
5	32.1, CH_2_	3.24 (d, 17.6) 2.14 (dd, 17.6, 6.4)	29.4, CH_2_	2.25 (td, 13.8, 3.0) 1.65 (m)	27.3, CH_2_	1.98 (m) 1.79 (m)
6	111.6, CH	5.75 (brd, 6.4)	26.8, CH_2_	2.40 (m) 1.97 (td, 14.4, 3.0)	29.4, CH_2_	2.23 (m) 1.62 (m)
7	144.9, C		97.0, C		96.7, C	
9	84.9, CH	4.23 (s)	78.0, CH	4.08 (s)	77.9, CH	4.01 (s)
10	196.9, C		199.8, C		199.7, C	
11	116.7, CH	6.03 (s)	121.5, CH	6.03 (s)	121.9, CH	5.82 (s)
12	154.2, C		160.8, C		160.8, C	
13	75.8, C		140.9, C		140.3, C	
14	32.7, CH_2_	1.93 (m)	132.1, CH	6.11 (dd, 5.4, 2.4)	128.8, CH	5.86 (m)
15	21.1, CH_2_	1.81 (m)	27.3, CH_2_	2.38 (m)	32.1, CH_2_	2.15 (dt, 18.0, 5.4) 2.05 (m)
16	49.2, CH	2.75 (m)	45.1, CH	2.96 (m)	32.2, CH	2.89 (m)
17	27.3, CH_2_	2.72 (m) 2.43 (td, 12.8, 2.8)	26.9, CH_2_	2.84 (dd, 13.2, 7.2) 2.45 (dd, 13.2, 10.2)	47.8, CH_2_	3.12 (dd, 17.4, 4.8) 2.50 (m)
18	118.5, C		119.5, C		203.7, C	
19	125.2, C		125.1, C		132.1, C	
20	110.4, CH	7.21 (s)	110.6, CH	7.23 (s)	120.9, CH	7.48 (s)
21	136.9, C		137.2, C		142.8, C	
22	121.4, CH	6.38 (d, 1.6)	121.5, CH	6.39 (d, 1.8)	119.8, CH	6.41 (d, 1.2)
23	141.8, C		142.0, C		145.5, C	
24	73.2, C		73.4, C		73.1, C	
26	72.8, C		72.9, C		72.9, C	
27	129.0, CH	6.47 (t, 1.6)	129.3, CH	6.51 (d, 1.8)	137.2, CH	6.77 (d, 1.2)
28	133.9, C		134.1, C		132.8, C	
29	128.8, C		129.3, C		137.9, C	
30	104.1, CH	7.43 (s)	104.2, CH	7.45 (s)	119.5, CH	7.18 (s)
31	137.9, C		138.5, C		135.9, C	
32	16.3, CH_3_	1.35 (s)	15.0, CH_3_	1.01 (s)	15.7, CH_3_	1.36 (s)
33	21.0, CH_3_	1.04 (s)	23.5, CH_3_	1.23 (s)	24.7, CH_3_	1.14 (s)
34	73.8, C		72.7, C		72.6, C	
35	25.2, CH_3_	1.28 (s)	24.3, CH_3_	1.28 (s)	24.2, CH_3_	1.20 (s)
36	26.5, CH_3_	1.33 (s)	26.9, CH_3_	1.34 (s)	26.8, CH_3_	1.29 (s)
37	31.5, CH_3_	1.54 (s)	31.7, CH_3_	1.55 (s)	31.7, CH_3_	1.53 (s)
38	31.4, CH_3_	1.54 (s)	31.6, CH_3_	1.54 (s)	31.6, CH_3_	1.52 (s)
39	30.8, CH_3_	1.46 (s)	31.0, CH_3_	1.47 (s)	31.0, CH_3_	1.46 (s)
40	30.9, CH_3_	1.46 (s)	31.0, CH_3_	1.47 (s)	31.0, CH_3_	1.46 (s)
7-OCH_3_			49.1, CH_3_	3.35 (s)	49.1, CH_3_	3.29 (s)

^a^At 400 (^1^H) and 100 (^13^C) MHz

^b^At 600 (^1^H) and 150 (^13^C) MHz

### Shearinine S (2)

Brown oil. [*α*] ^20^_D_ + 15 (*c* = 0.10, MeOH). UV (MeOH) *λ*_max_ (log *ε*) 368 (0.3), 282 (2.7), 215 (1.3) nm. ECD (0.25 mmol/L, MeOH) *λ*_max_ (Δ*ε*) 353 (− 13.6), 280 (− 61.4), 235 (+ 26.2), 215 (− 15.2) nm. ^1^H NMR (CDCl_3_, 600 MHz) and ^13^C NMR (CDCl_3_, 150 MHz), see Table 1; HRESIMS *m/z* 618.3187, [M + Na]^+^, (calcd. for C_38_H_45_NNaO_5_^+^, 618.3190).

### Antibacterial assays

Nine bacterial strains, including Gram-positive *Staphylococcus aureus* (ATCC 43300), *S. aureus* (ATCC 33591), *S. aureus* (ATCC 29213), *S. aureus* (ATCC 25923), *Enterococcus faecalis* (ATCC 51299), *E. faecium* (ATCC 35667), and *Bacillus subtilis* (ATCC 19659) and Gram-negative *Escherichia coli* (ATCC 25922) and *Vibrio parahaemolyticus* (ATCC 17802) were used for the antibacterial assay. The specific antibacterial assay was carried out as described previously (Verma et al. 2022). Vancomycin·HCl was used as a positive control.

### Cytotoxicity assays

The cytotoxic activity was evaluated against the human hepatocellular carcinomas cells HepG2 and human gastric cancer cells SGC-7901 by the MTT method as described previously (Mosmann 1983). Cisplatinum was used as a positive control with IC_50_ values of 1.99 and 3.56 μg/mL, respectively.

## Results and discussion

**Fig. 1:**
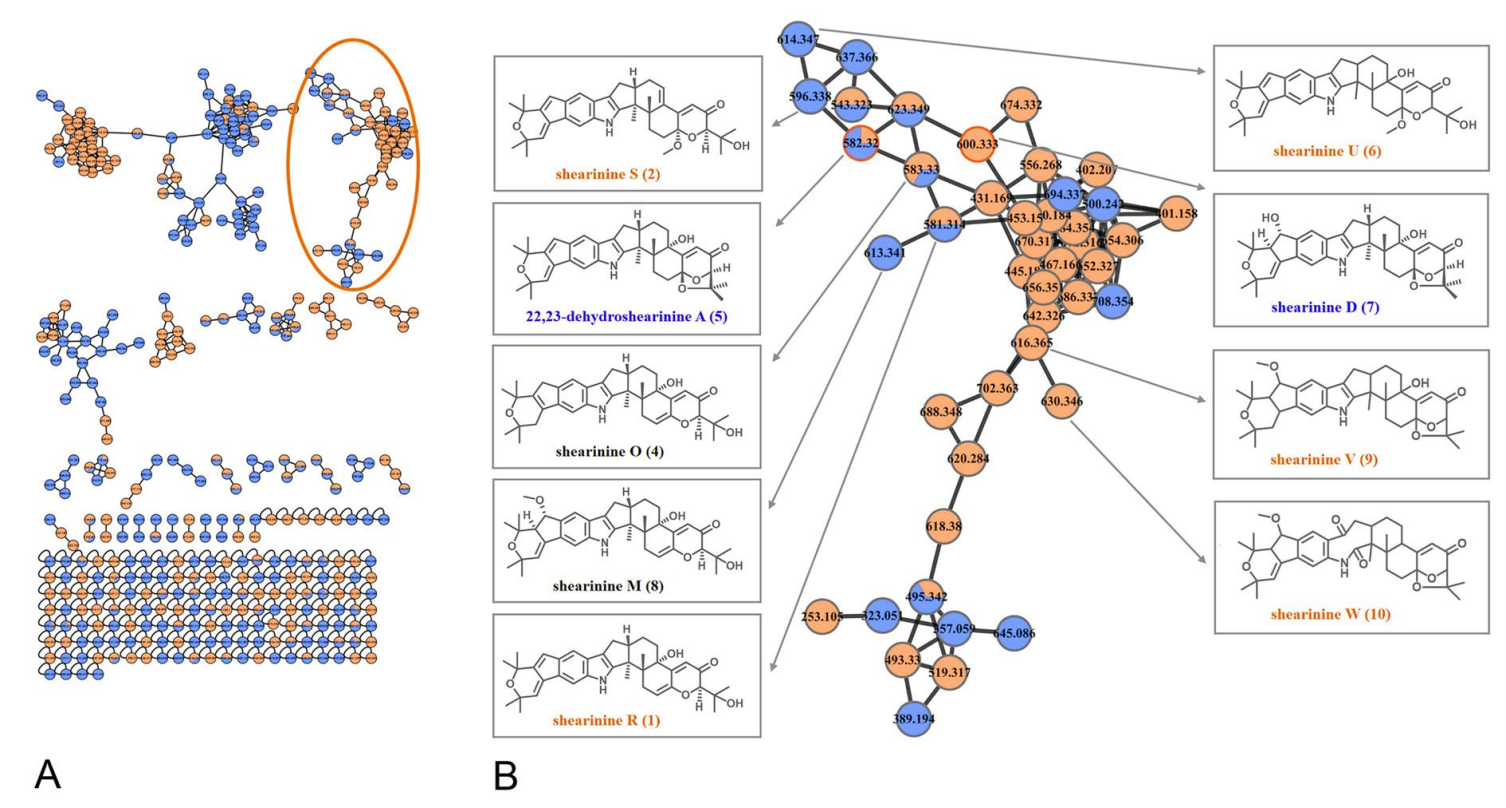
Molecular network generated from two fractions of the fungal extracts from *Penicillium* sp. N4-3 (orange nodes from Fr. 1 and purple nodes from Fr. 2). The cluster of related ions corresponding to indole diterpenoids highlighted is enlarged on the left. The two indole diterpenoids annotated by the GNPS database are named in blue. The other predicted compounds by molecular networks are named in orange (new) and black (known)

**Fig. 2:**
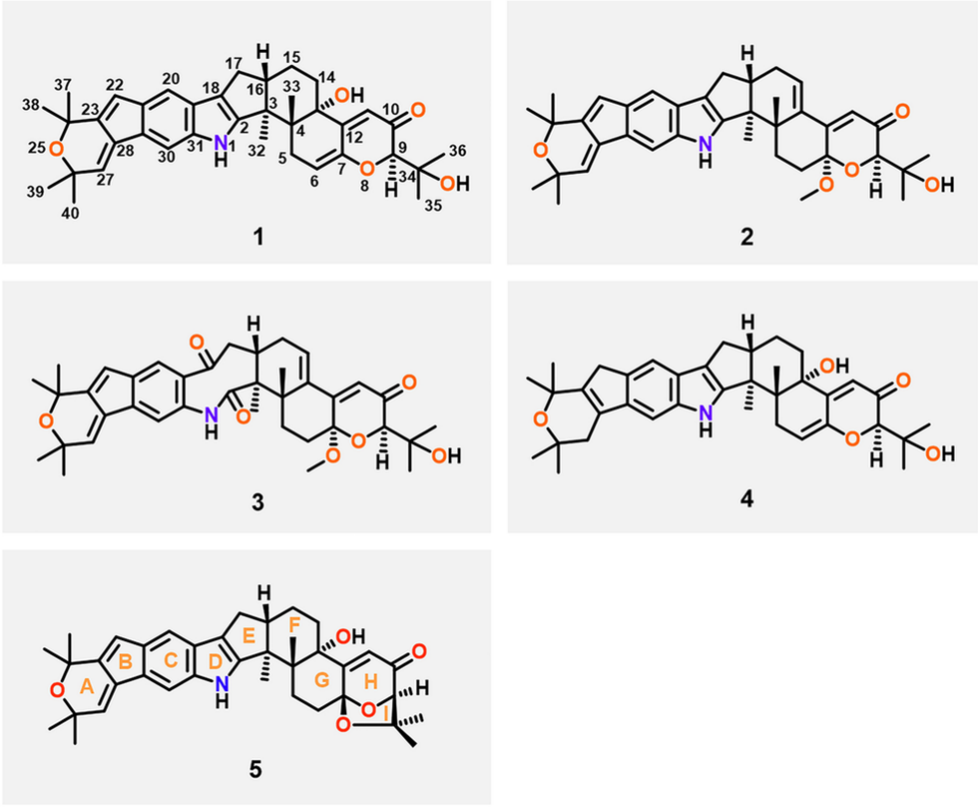
The structures of the isolated indole diterpenoids (**1**−**5**)

The marine-derived fungus *Penicillium* sp. N4-3 was cultured on potato dextrose broth (PDB) medium at 25 °C without shaking for 1 month. The broth and mycelia extracts of this fungus were pretreated by silica gel column chromatography to remove the small polar oil components and the large polar sugars and salts, and then two fractions (Fr.1 and Fr.2) were taken to highlight the characteristic metabolites. Hence, these two fractions were subjected to HPLC–MS/MS analysis, and a comprehensive molecular network was constructed with the converted MS/MS data (Fig. S35). Two nodes were putatively identified as 22,23-dehydroshearinine A (**5**, the ion at* m*/*z* 582) and shearinine D (**7**, the ion at* m*/*z* 600) by matching with the GNPS molecular library, which highlighted the “molecular family” of indole diterpenoids in the molecular network (Fig. 1). Upon confirmation of this molecular scaffold, a group of potential indole diterpenoids could be proposed in this cluster depending on GNPS (Fig. 1). Mass-based chemical investigation of Fr.1 and Fr.2 led to the targeted isolation of two new indole diterpenoids, shearinines R and S (**1**, **2**), together with two known analogs, shearinine O (**4**) (Ariantari et al. 2019) and 22,23-dehydroshearinine A (**5**) (You et al. 2013) (Fig. 2). An artifact, shearinine T (**3**), produced by the automatic oxidation of **2** was also described. Furthermore, a suite of five indole diterpenoids (**6**−**10**), including three new ones, shearinines U−W (**6**, **9**, **10**), in this network were predicted the combination of GNPS molecular networking and manual analysis of MS/MS fragmentation clusters.

**Fig. 3:**
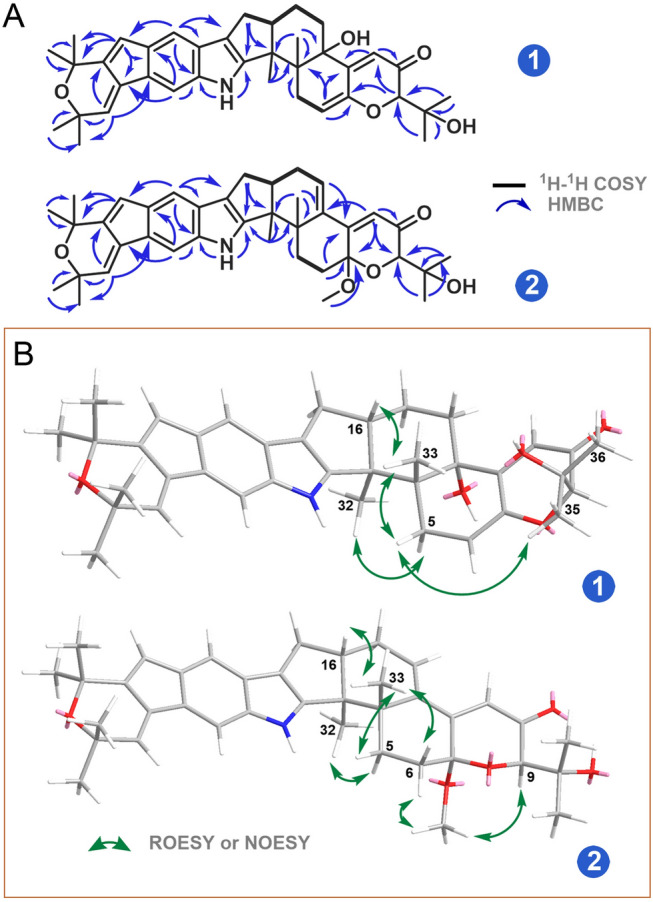
**A** Key ^1^H-^1^H COSY and HMBC correlations of **1** and **2**; **B** key ROESY or NOESY correlations of **1** and **2**

Shearinine R (**1**) was isolated as a brown oil. Its molecular formula of C_37_H_43_NO_5_ was determined based on HRESIMS spectrum, indicating 17 degrees of unsaturation and a mass of 604.3042 [M + Na]^+^ (calcd. for C_37_H_43_NNaO_5_^+^, 604.3033), 2 Da less than that of the well-described indole triterpenoid shearinine O (**4**) (Ariantari et al. 2019). The IR absorptions at 3329 (br) and 1658 cm^−1^ suggested the presence of hydroxyl and carbonyl groups. The ^13^C NMR spectrum (Table 1) displayed all 37 carbons, corresponding to 8 methyls, 4 methylenes, 8 methines, and 17 quaternary carbons including one carbonyl group (*δ*_C_ 196.9, C-10). Investigation of the ^1^H NMR data (Table 1) revealed the presence of one D_2_O exchangeable proton at *δ*_H_ 7.81 (brd, *J* = 6.4 Hz) for an indole amine, eight aliphatic methyl singlets at *δ*_H_ 1.35 (H_3_-32), 1.04 (H_3_-33), 1.28 (H_3_-35), 1.33 (H_3_-36), 1.54 (H_3_-37), 1.54 (H_3_-38), 1.46 (H_3_-39), and 1.46 (H_3_-40), four sets of methylene signals, eight methines including two aromatic singlets at *δ*_H_ 7.21 (H-20) and 7.43 (H-30), four olefinic signals at *δ*_H_ 5.75 (brd, *J* = 6.4 Hz, H-6), 6.03 (s, H-11), 6.38 (d, *J* = 1.6 Hz, H-22), and 6.47 (t, *J* = 1.6 Hz, H-27), and two further aliphatic protons. The HSQC spectrum allowed to assign all protons to their corresponding carbon atoms. The ^1^H and ^13^C NMR data, in combination with the HMBC correlation analysis, confirmed the assignments of 8 rings for **1**, which was in accordance with that of shearinine O (**4**) (Ariantari et al. 2019). The main difference between **1** and **4** was found in Rings A and B where the double bond between C-23 and C-28 of **4** was replaced by two double bonds at C-22−C-23 and C-27−C-28 in **1**. The HMBC correlations (Fig. 3A) from H-22 to C-20, C-24, C-28, and C-29, and from H-27 to C-23, C-26, C-29, and C-40 confirmed this substitution for **1**. Therefore, the planar structure of **1** was unambiguously established.

The relative configuration of** 1** was established by analysis of its ROESY spectrum (Fig. 3B). The observed key ROESY correlations from H*β*-5 to H_3_-33/H_3_-35 and from H_3_-33 to H-16 indicated the co-facial orientation of these protons. Meanwhile, the ROESY correlation between H*α*-5 and H_3_-32 assigned the opposite face of these protons. The absolute configuration of **1** was determined by ECD spectrum (Supplementary Fig. S8). The ECD data of **1** showed the negative (373 nm, 281 nm) and positive (324 nm, 251 nm) Cotton effects, which matched well with those of the very similar compound shearinine O (**4**). The latter shows a 3*S*,4*S*,9*R*,16*S*-configuration (Ariantari et al. 2019), which allowed the assignment of 3*S*,4*S*,9*R*,16*S*-configuration for **1**.

Shearinine S (**2**) was also isolated as a brown oil with the molecular formula of C_38_H_45_NO_5_ determined by HRESIMS spectrum. Careful analysis of the ^1^H and ^13^C NMR spectroscopic data (Table 1) of **2** revealed structural similarity to that of **1**, except for the presence of a methoxy group (*δ*_C_ 49.1, *δ*_H_ 3.35, 7-OCH_3_) in **2** and an olefinic proton signal shifted slightly to the downfield (*δ*_H_ 6.11 (H-14) in **2** vs *δ*_H_ 5.75 (H-6) in **1**). In the HMBC spectrum (Fig. 3A), the correlations from 7-OCH_3_ to C-7 (*δ*_C_ 97.0) revealed this group was connected to C-7, as well as the HMBC correlations from H-6, H-9, and H-11 to C-7 further confirmed this assignment. In addition, the HMBC correlations from H-14 to C-4, C-12, and C-16, and from H-11 to C-13 and the ^1^H-^1^H COSY correlation between H-14 and H-15 established a double bond between C-13 and C-14. The cross-peak of 7-OCH_3_ and H-9 in the NOESY spectrum (Fig. 3B) indicated the *α*-orientation for 7-OCH_3_. Moreover, the NOESY correlation between H-16 and H_3_-33 implied a *trans*-3,16-ring junction as observed in former congeners. The ECD data of **2** (Supplementary Fig. S16) matched well with those of 7-hydroxypaxilline-13-ene (Ariantari et al. 2019), which confirmed the 3*S*,4*S*,7*S*,9*R*,16*S*-configuration for **2**.

Interestingly, a series of new signals were observed in chloroform solution during NMR measurement of compound **2** (Figs. S17−18). Careful analysis of the emerging NMR signals (Table 1) showed that it corresponded to the autoxidation product from **2** by oxidization and cleavage of the indole moiety at the C–2−C–18 bond. Similar automatic oxidation of indole diterpenoids has also been mentioned in the literature (Xu et al. 2007). This new artifact was named as shearinine T (**3**).

**Fig. 4:**
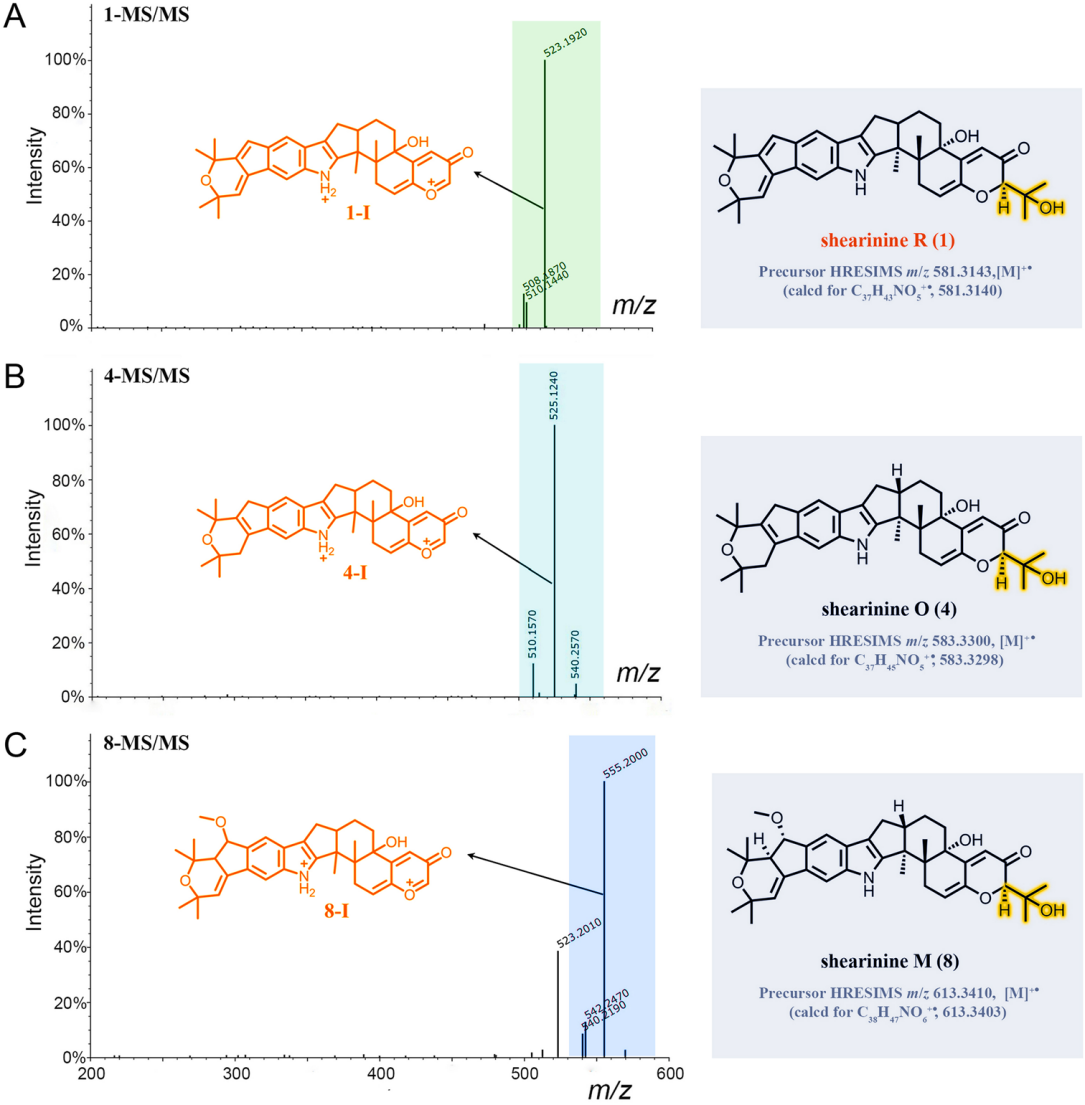
MS/MS spectra of the [M]^+•^ ions at *m*/*z* 581.3143 (shearinine R (**1**)) (**A**), 583.3300 (shearinine O (**4**)) (**B**), and 613.3410 (shearinine M (**8**)) (**C**). The proposed type I signature ions are shown

**Fig. 5:**
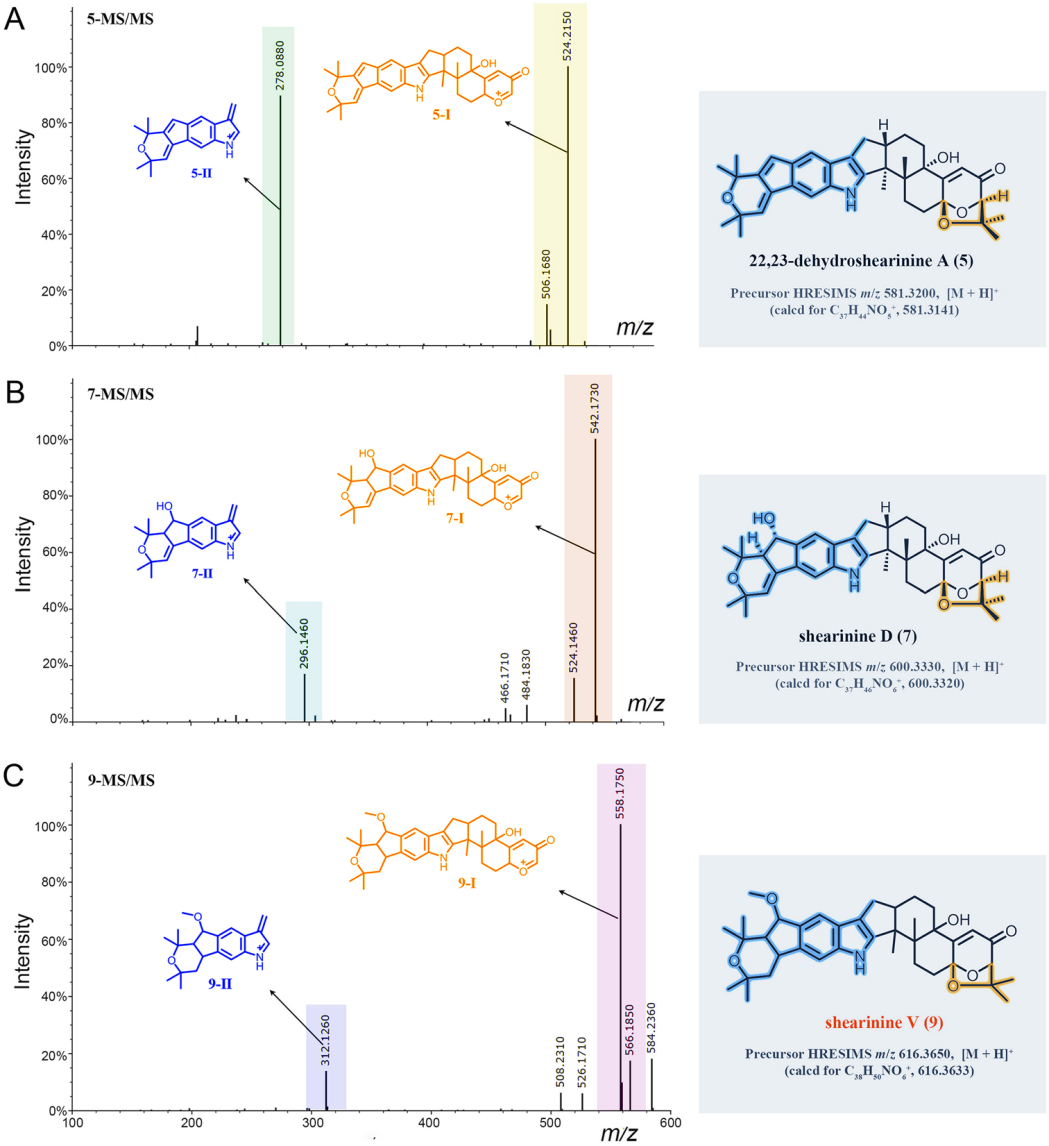
MS/MS spectra of the [M + H]^+^ ions at *m*/*z* 581.3200 (22,23-dehydroshearinine A (**5**)) (**A**), 600.3330 (shearinine D (**7**)) (**B**), and 616.3650 (shearinine V (**9**)) (**C**). The proposed type I and II signature ions are shown

**Fig. 6:**
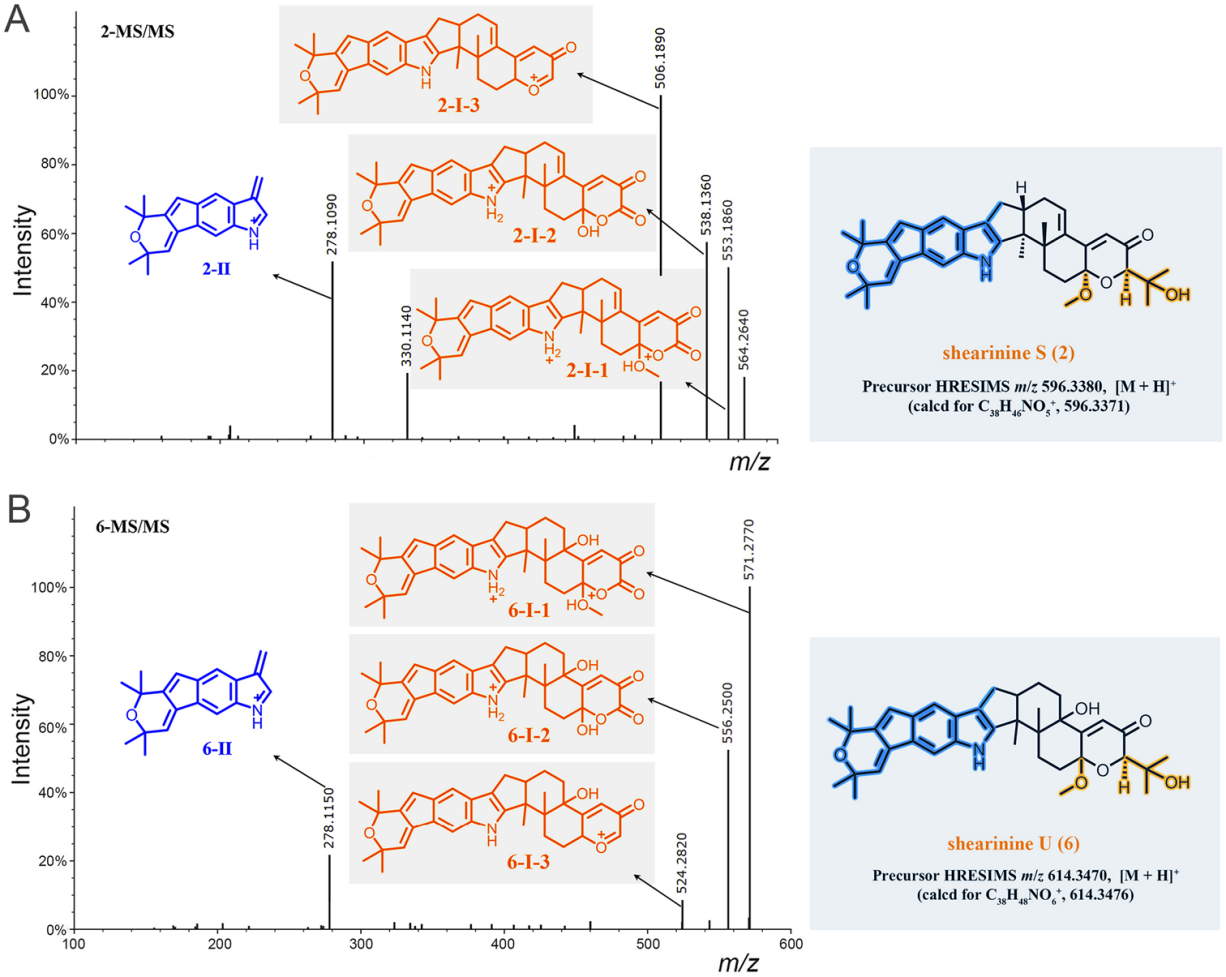
MS/MS spectra of the [M + H]^+^ ions at *m*/*z* 596.3380 (shearinine S (**2**)) (**A**) and 614.3470 (shearinine U (**6**)) (**B**). The proposed type I and II signature ions are shown

**Fig. 7:**
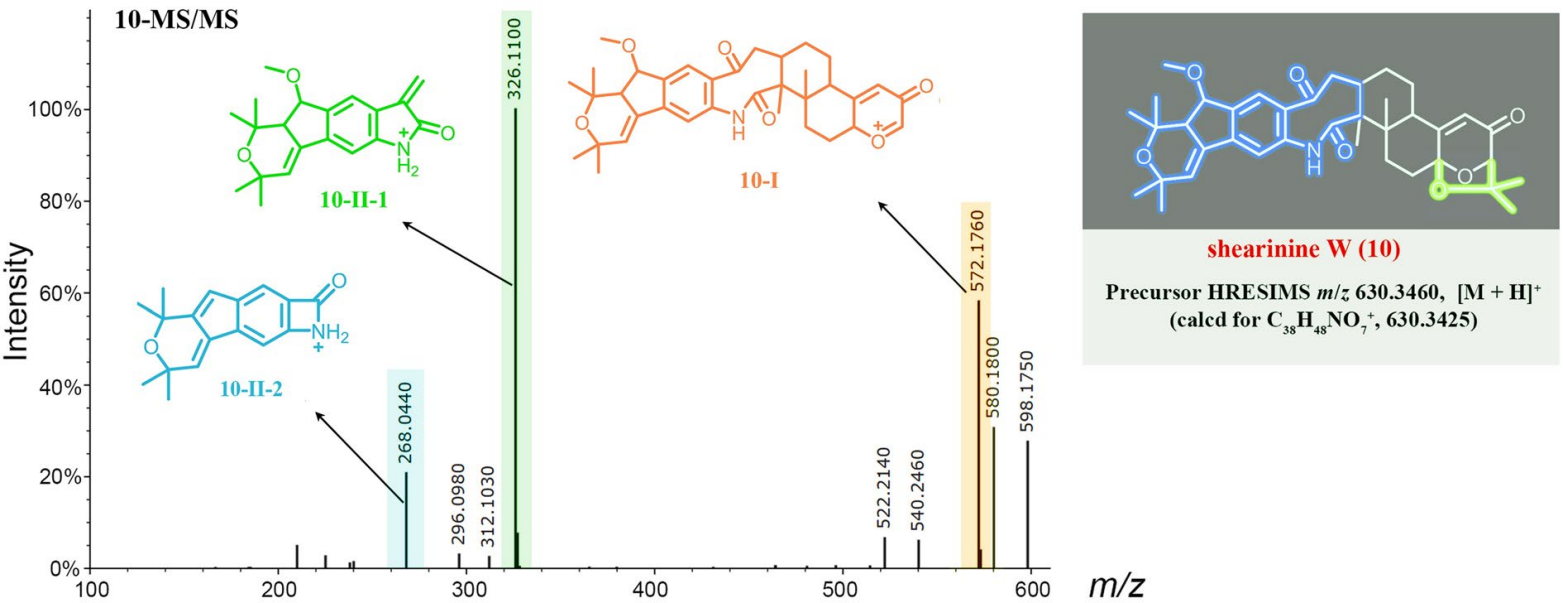
MS/MS spectrum of the [M + H]^+^ ion at *m*/*z* 630.3460 (shearinine W (**10**)). The proposed type I and II signature ions are shown

Besides the isolated compounds (**1**, **2**, **4**,** 5**) and shearinine D (**7**) annotated by GNPS, many other putatively new or known indole diterpenoids were located in the molecular ion cluster (Fig. 1). Ultimately, based on careful manual analysis of the MS/MS spectra (Table 2, Figs. 4, 5, 6, 7, Supplementary Figs. S19−27), four other indole diterpenoids (**6**,** 8**−**10**) including three new ones named as shearinines U−W (**6**, **9**, **10**) were identified as shown in Fig. 1. It should be noted that compound **3** does not appeared in the cluster, suggesting that the production of **3** does not involve biosynthetic enzymes.

**Table 2:** Observed MS/MS fragmentation ions of shearinine derivatives

Compounds	Chemical formula	MS (*m*/*z*)	MS/MS (*m*/*z*)		
			Type I	Type II	Type III
Shearinine R (**1**)	C_37_H_43_NO_5_	581 [M]^+•^	523, 508		510
Shearinine S (**2**)	C_38_H_45_NO_5_	596 [M + H]^+^	553, 538, 506,	278	564, 330
Shearinine O (**4**)	C_37_H_45_NO_5_	583 [M]^+•^	540, 525, 510		
22,23-Dehydroshearinine A (**5**)	C_37_H_43_NO_5_	582 [M + H]^+^	524, 506	278	
Shearinine U (**6**)	C_38_H_47_NO_6_	614 [M + H]^+^	571, 556, 524	278	
Shearinine D (**7**)	C_37_H_45_NO_6_	600 [M + H]^+^	542, 524	296	484, 466
Shearinine M (**8**)	C_38_H_47_NO_6_	613 [M]^+•^	555, 540, 523		542
Shearinine V (**9**)	C_38_H_49_NO_6_	616 [M + H]^+^	558, 526, 508	312	584, 566
Shearinine W (**10**)	C_38_H_47_NO_7_	630 [M + H]^+^	572, 540	326, 312, 296, 268	598, 580

Manual analysis of characteristic fragmentation clusters confirmed that there were two types of signature ions present in the MS/MS spectra of these indole diterpenoids (Table 2, Figs. 4, 5, 6, 7). Type I was the dominant signals produced by breakage at C-9 side chain or Ring I, while Type II was the conserved fragments related to the indole moiety which was relatively stable. The abundance of Type II ions was lower than that of Type I ions except for compound **10**. The signals about the breaking of Rings F, G, and H were relatively weak, almost no signal in many MS/MS spectra. In addition, other low abundance ions associated with dehydration or demethoxylation could also be found in some MS/MS spectra. These low abundance or accidentally generated ions were classified as Type III ions.

The above MS/MS characteristic fragment ions provided clues for the structure elucidation of these indole diterpenoids. Type II directly indicated the structural unit of Rings A–D for compounds **2**, **5–7**, and **9** (Figs. 5, 6), while Type I can distinguish whether the compound possessed a C-9 side chain (**1**, **4**, and **8**, the feature fragment with a double bond at C_6_-C_7_, Fig. 4) or a Ring I (**5**, **7**,** 9**, and **10**, the feature fragment with a single bond at C_6_–C_7_, Figs. 5 and 7). If a methoxy group was connected to C-7 (**2** and **6**, Fig. 6), then Type I may appear in three forms of feature fragment ions. The first was formed by oxidative break of C-9 side chain only (*m*/*z* 553, **2**-I-1; *m*/*z* 571, **6**-I-1); the second was loss of methyl group at the same time (*m*/*z* 538, **2**-I-2;* m*/*z* 556, **6**-I-2), and the third was complete loss of methoxy group at the same time to form the feature fragment with a C_6_–C_7_ single bond (*m*/*z* 506, **2**-I-3; *m*/*z* 524, **6**-I-3). Distinctively, compounds **1**, **4**, and **8** produced high abundance of Type I ions, but the abundance of Type II ions was low and almost completely disappeared (Fig. 4). The reasons may be due to the presence of a similar large unsaturated heterocyclic system in their structures, forming parent ions that lose only one electron in the primary mass spectra.

It is worth noting that **10** was the outermost node in the molecular family (Fig. 1), which illustrated that the structure of **10** may be significantly different from other compounds. On account of the molecular formula and MS/MS characteristic fragment ions of **10** (Fig. 7), it could be inferred that **10** had no hydroxyl group at C-13. First, the Type I ion at *m*/*z* 572 (**10**-I) was formed by I-ring fracture, which are consistent with those of Type I ions (*m*/*z* 524, 542, 558) shown in Fig. 5. Specially, **10** possessed the unstable unit of C-2, C-18 dicarbonyl eight-member cyclolactam, which increased the probability of a break in this ring, and showed a distinctive MS/MS spectrum (Fig. 7). For example, the eight-member ring was prone to fracture at many bonds, forming a very special Type II ion at *m*/*z* 268 (**10-II-**2), and a dominant Type II ion at *m*/*z* 326 (**10**-II-1) with higher abundance than that of Type I ion at *m*/*z* 572 (**10**-I).

From the perspective of biosynthesis, the three new structures predicted by molecular networks, shearinines U−W (**6**, **9**, **10**), were closely related to the isolated indole diterpenoids (**1**−**5**). Shearinine U (**6**) was the 13-OH precursor of shearinine S (**2**), i.e., **2** was the dehydrated product from** 6**. Therefore, **6** and **2** formed mutual neighboring nodes in the molecular ion cluster due to structural similarity. In addition, **6** can be rationally deemed as the ring opening methylation product from the known compound 22,23-dehydroshearinine A (**5**). Shearinine V (**9**) was also a derivative formed by the C-27 double bond reduction, and C-22 double bond oxidation and methylation from **5**. Similar to shearinine T (**3**), shearinine W (**10**) possessed a special oxidative cleavage of the C-2-C-18 double bond, which was common in the indole diterpenoid family (Ariantari et al. 2019; Xu et al. 2007).

The above well-elucidated 9 nodes were networked to other unknown features in the molecular family containing 44 nodes, suggesting the discovery of an entire suite of structurally related compounds. However, due to the varied and complex of chemical structure and fracture manner of indole diterpenoids, other nodes in the cluster have not been successfully predicted. To crack the structures of these nodes, a more practical approach is to increase the yields of these compounds by metabolic regulation strategies, and then isolate and characterize them. These efforts are currently under way.

Compounds **1**, **4**, and **5** with relatively high yields were tested for their antibacterial activity against a panel of pathogenic bacteria. The results (Table 3) indicated that compound** 1** showed antibacterial activities against seven strains of tested bacteria, including both antibiotic-resistant and -susceptible strains with IC_50_ values ranging from 6.34 to 47.96 μg/mL. Compound **5** instead exhibited no activity against the tested bacteria, which suggested that the cleavage of ring I may play a critical role in the antibacterial activities. Furthermore, compound **1** showed cytotoxicity against human hepatocellular carcinomas cells HepG2 and human gastric cancer cell line SGC-7901 with IC_50_ values of 6.27 and 19.16 μg/mL, respectively.

**Table 3:** Antibacterial activities of compounds **1**, **4** and **5**^*a*^

Pathogenic bacteria	IC_50_ (*μ*g/mL, $\overline{{\text{x}}}$ ± s, n = 3)				
	**1**	**4**	**5**	O^b^	V^c^
*S. aureus* ATCC 43300	47.96 ± 4.42	63.53 ± 9.97	> 100	22.41 ± 11.47	1.78 ± 0.22
*S. aureus* ATCC 33591	27.58 ± 2.25	> 100	> 100	> 100	1.12 ± 0.11
*S. aureus* ATCC 25923	23.68 ± 0.41	> 100	> 100	0.029 ± 0.05	1.64 ± 0.01
*S. aureus* ATCC 29213	23.25 ± 2.23	> 100	> 100	0.05 ± 0.03	1.62 ± 0.72
*E. faecium* ATCC 51299	32.89 ± 8.80	> 100	> 100	2.37 ± 1.22	5.82 ± 5.33
*E. faecium* ATCC 35667	> 100	> 100	> 100	8.68 ± 2.85	0.68 ± 0.12
*E. coli* ATCC 25922	> 100	> 100	> 100	> 100	> 100
*B. subtilis* ATCC 19659	22.20 ± 7.36	37.90 ± 11.15	> 100	0.15 ± 0.07	0.35 ± 0.01
*V. parahaemolyticus* ATCC 17802	6.48 ± 1.61	20.24 ± 4.39	> 100	> 100	1.29 ± 0.18

^a^Data represent IC_50_ values. ^*b*^O = oxacillin sodium. ^*c*^V = Vancomycin HCl

## Conclusion

In this work, molecular networking was efficiently applied to rapidly dereplicate known compounds and explore related secondary metabolites of the same structural class from a marine-derived fungus. Using MS/MS-based molecular networks generated with GNPS, guided isolation of the two new indole diterpenoids shearinines R and S (**1**, **2**), a new oxidative artifact shearinine T (**3**), together with two known analogs (**4**, **5**), from the marine-derived fungus *Penicillium* sp. N4-3. Furthermore, five related shearinine derivatives (**6**−**10**) were identified from the network by manual analyzing and comparing the respective MS/MS spectra. The main abundant ionic species in the MS/MS spectra of shearinines revealed two types of feature fragments associated with the indole moiety and the breaking of C-9 side chain or Ring I. Compound **1** showed significant antibacterial and cytotoxic activity.

## Supplementary Information

Below is the link to the electronic supplementary material.

Supplementary file1 (DOCX 15274 KB)

## Data Availability

Data of all figures in this paper are available from the Supporting Information.
