# Supplementary material for: Molecular networking reveals indole diterpenoids from the marine-derived fungus Penicillium sp. N4-3
Source: Mar Life Sci Technol. 2025 Apr 7;7(2):302–12. doi: 10.1007/s42995-024-00274-6 (PMC12102448; doi:10.1007/s42995-024-00274-6)
Supplement: Supplementary file 1 — Supplementary file1 (DOCX 15274 KB) [file 42995_2024_274_MOESM1_ESM.docx]

***Supplementary Information***

**Molecular networking reveals indole diterpenoids from the marine-derived fungus *Penicillium* sp. N4-3**

Min Chen^1,†,^*, Bao-Cong Hao^1,†^, Xia-Hao Zhu^1^, Li-Kui Zhang^1^, Yao-Yao Zheng^2,3^, Xiao-Jian Zhou^1^, Till F. Schäberle^4,5,6^, Li Shen^7^, Chang-Yun Wang^2,3,^* and Yang Liu^4,5,^*

*^1^ Marine Science & Technology Institute, College of Environmental Science & Engineering, Yangzhou University, 196#, Huayang West Street, Yangzhou 225127, People’s Republic of China*

*^2^ Key Laboratory of Marine Drugs, the Ministry of Education of China, Institute of Evolution & Marine Biodiversity, School of Medicine and Pharmacy, Ocean University of China, Qingdao 266003, People’s Republic of China*

*^3^Laboratory for Marine Drugs and Bioproducts, Qingdao National Laboratory for Marine Science and Technology, Qingdao 266237, People’s Republic of China*

*^4^ Institute for Insect Biotechnology, Justus-Liebig-University Giessen, 35392 Giessen, Germany*

*^5^ Fraunhofer Institute for Molecular Biology and Applied Ecology (IME), Branch for Bioresources, 35392 Giessen, Germany*

*^6^German Center for Infection Research (DZIF), Partner Site Giessen-Marburg-Langen, 35392 Giessen, Germany*

*^7^Institute of Transla tional Medicine, Medical College, Yangzhou University Yangzhou 225001, People’s Republic of China*

^†^ M. Chen and B. C. Hao contributed equally to this article.

* To whom correspondence should be addressed: E-mail: dieying0719@163.com (M. Chen); [Liu.Yang@agrar.uni-giessen.de](file:///\\ime.fraunhofer.de\ime\Homes\Giessen\michael.marner\PostDoc\PAPERS\contributions\Phenethylamine%20alkaloids\Liu.Yang@agrar.uni-giessen.de) (Y. Liu); changyun@ouc.edu.cn (C. Y. Wang).

**List of Supplementary Information**

**Figure S1.** ^1^H NMR (400 MHz, CDCl_3_) spectrum of compound **1**

**Figure S2.** ^13^C NMR (100 MHz, CDCl_3_) spectrum of compound **1**

**Figure S3.** HSQC (CDCl_3_) spectrum of compound **1**

**Figure S4.** ^1^H–^1^H COSY (CDCl_3_) spectrum of compound **1**

**Figure S5.** HMBC (CDCl_3_) spectrum of compound **1**

**Figure S6.** ROESY (CDCl_3_) spectrum of compound **1**

**Figure S7.** HRESIMS spectrum of compound **1**

**Figure S8**. ECD spectrum of **1**

**Figure S9.** ^1^H NMR (600 MHz, CDCl_3_) spectrum of compound **2**

**Figure S10.** ^13^C NMR (150 MHz, CDCl_3_) spectrum of compound **2**

**Figure S11.** HSQC (CDCl_3_) spectrum of compound **2**

**Figure S12.** ^1^H–^1^H COSY (CDCl_3_) spectrum of compound **2**

**Figure S13.** HMBC (CDCl_3_) spectrum of compound **2**

**Figure S14.** NOESY (CDCl_3_) spectrum of compound **2**

**Figure S15.** HRESIMS spectrum of compound **2**

**Figure S16**. ECD spectrum of **2**

**Figure S17.** ^1^H NMR (600 MHz, CDCl_3_) spectrum of compound **3**

**Figure S18.** ^13^C NMR (150 MHz, CDCl_3_) spectrum of compound **3**

**Figure S19.** (Upper) MS/MS spectrum of shearinine R (**1**); (Under) MS/MS fragment ions of shearinine R (**1**)

**Figure S20.** (Upper) MS/MS spectrum of shearinine S (**2**); (Under) MS/MS fragment ions of shearinine S (**2**)

**Figure S21.** (Upper) MS/MS spectrum of shearinine O (**4**); (Under) MS/MS fragment ions of shearinine O (**4**)

**Figure S22.** (Upper) MS/MS spectrum of 22,23-dehydroshearinine A (**5**); (Under) MS/MS fragment ions of 22,23-dehydroshearinine A (**5**)

**Figure S23.** (Upper) MS/MS spectrum of shearinine U (**6**); (Under) MS/MS fragment ions of shearinine U (**6**)

**Figure S24.** (Upper) MS/MS spectrum of shearinine D (**7**); (Under) MS/MS fragment ions of shearinine D (**7**)

**Figure S25.** (Upper) MS/MS spectrum of shearinine M (**8**); (Under) MS/MS fragment ions of shearinine M (**8**)

**Figure S26.** (Upper) MS/MS spectrum of shearinine V (**9**); (Under) MS/MS fragment ions of shearinine V (**9**)

**Figure S27.** (Upper) MS/MS spectrum of shearinine W (**10**); (Under) MS/MS fragment ions of shearinine W (**10**)

**Figure S28.** The thin layer chromatography analysis of the fungal extracts from *Penicillium* sp. N4-3

**Figure S29.** Full molecular networking of the profiled compounds from two fractions of the fungal extracts from *Penicillium* sp. N4-3 annotated by GNPS database

**Figure S30.** Phylogenetic tree of ITS rRNA sequences of closely related *Penicillium* sp. N4-3.


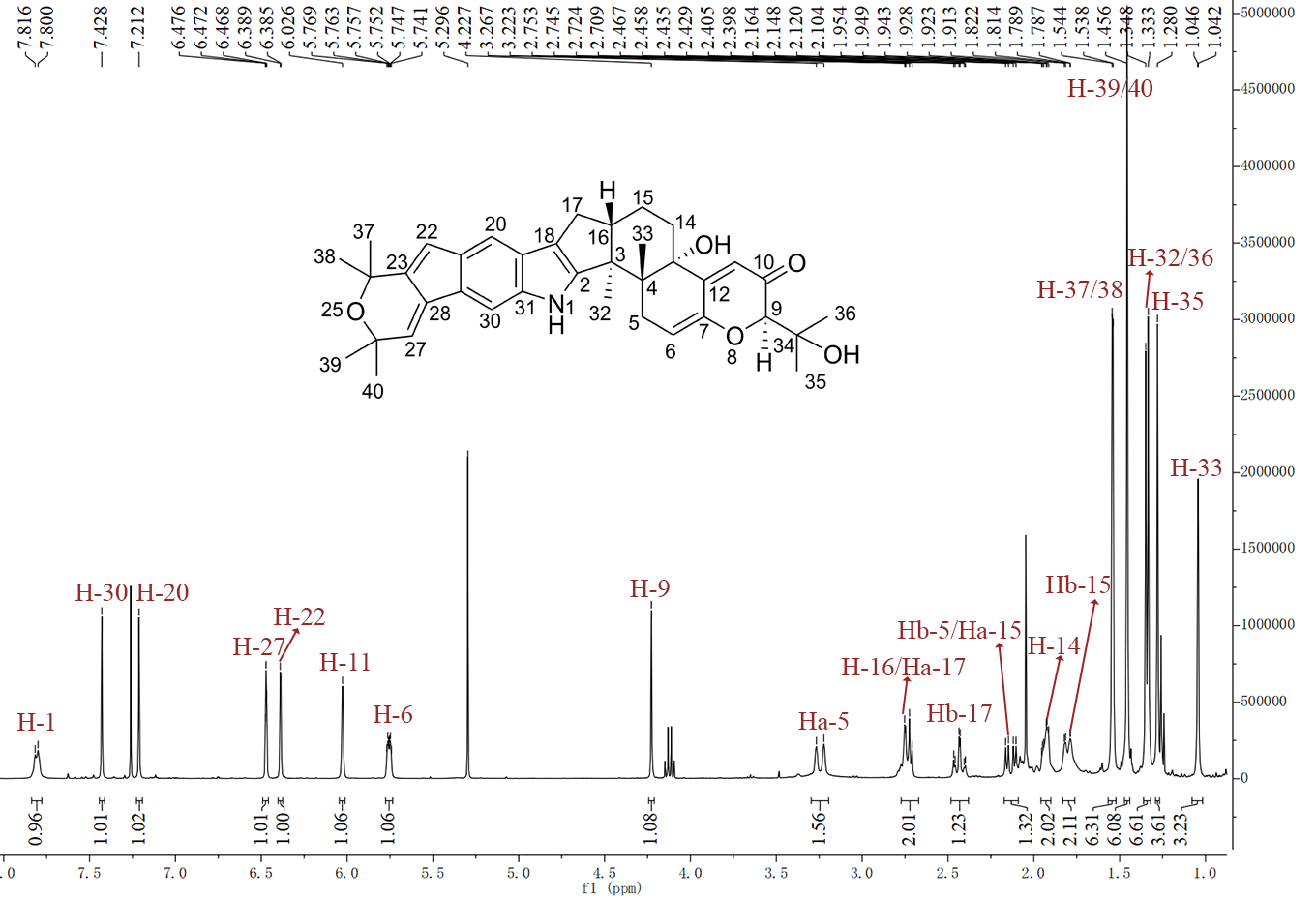


**Figure S1.** ^1^H NMR (400 MHz, CDCl_3_) spectrum of compound **1**


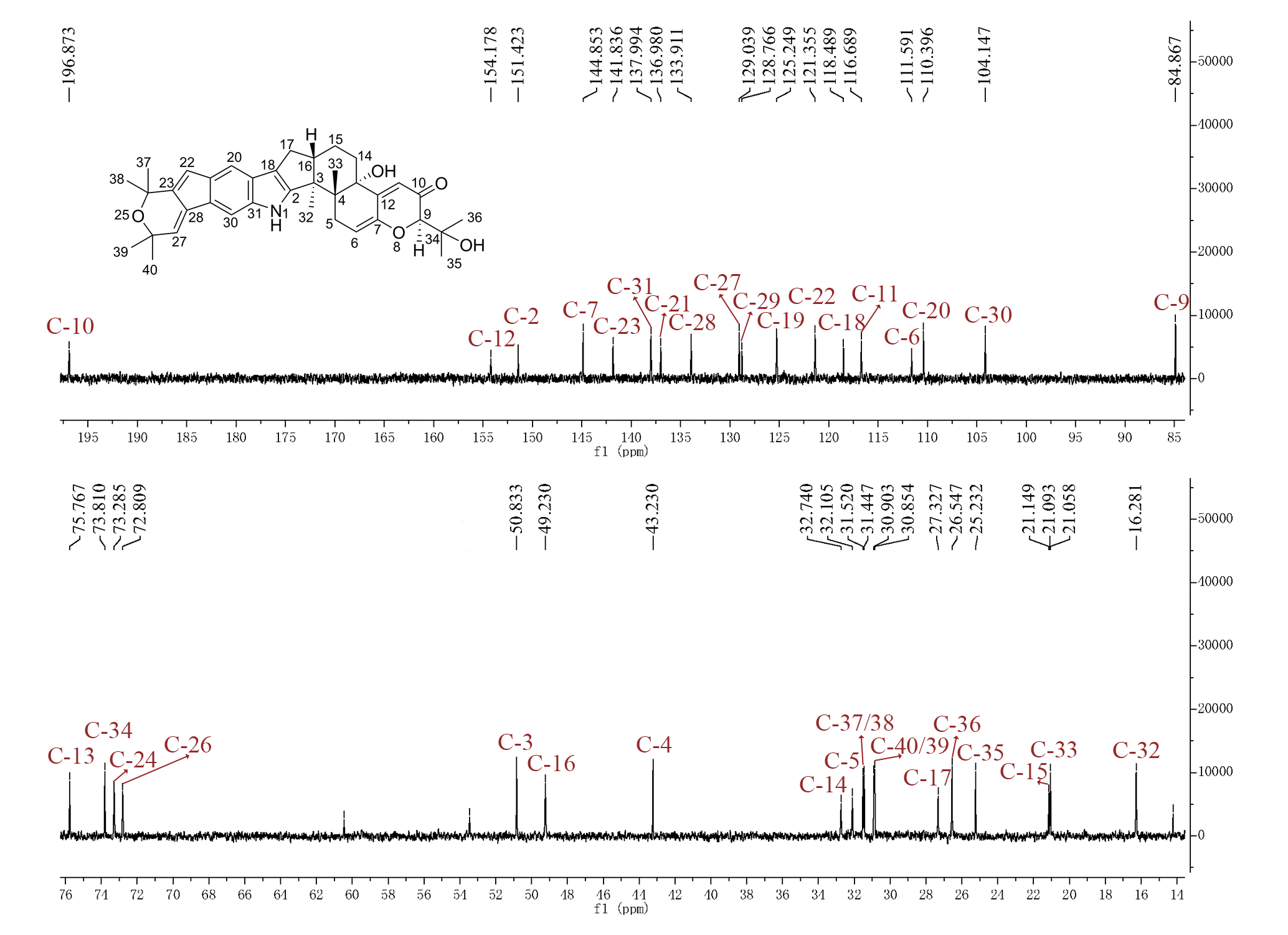


**Figure S2.** ^13^C NMR (100 MHz, CDCl_3_) spectrum of compound **1**


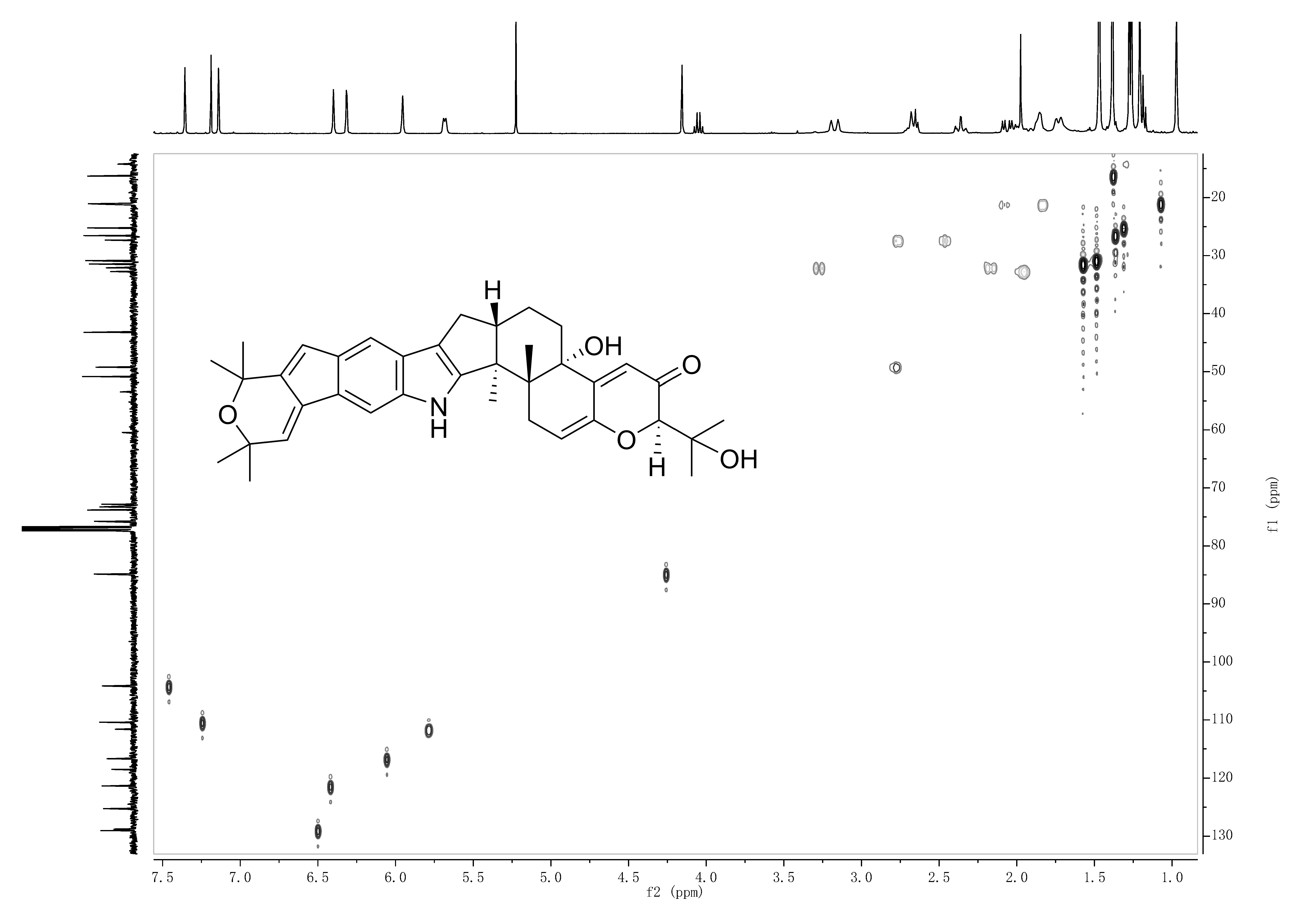


**Figure S3.** HSQC (CDCl_3_) spectrum of compound **1**


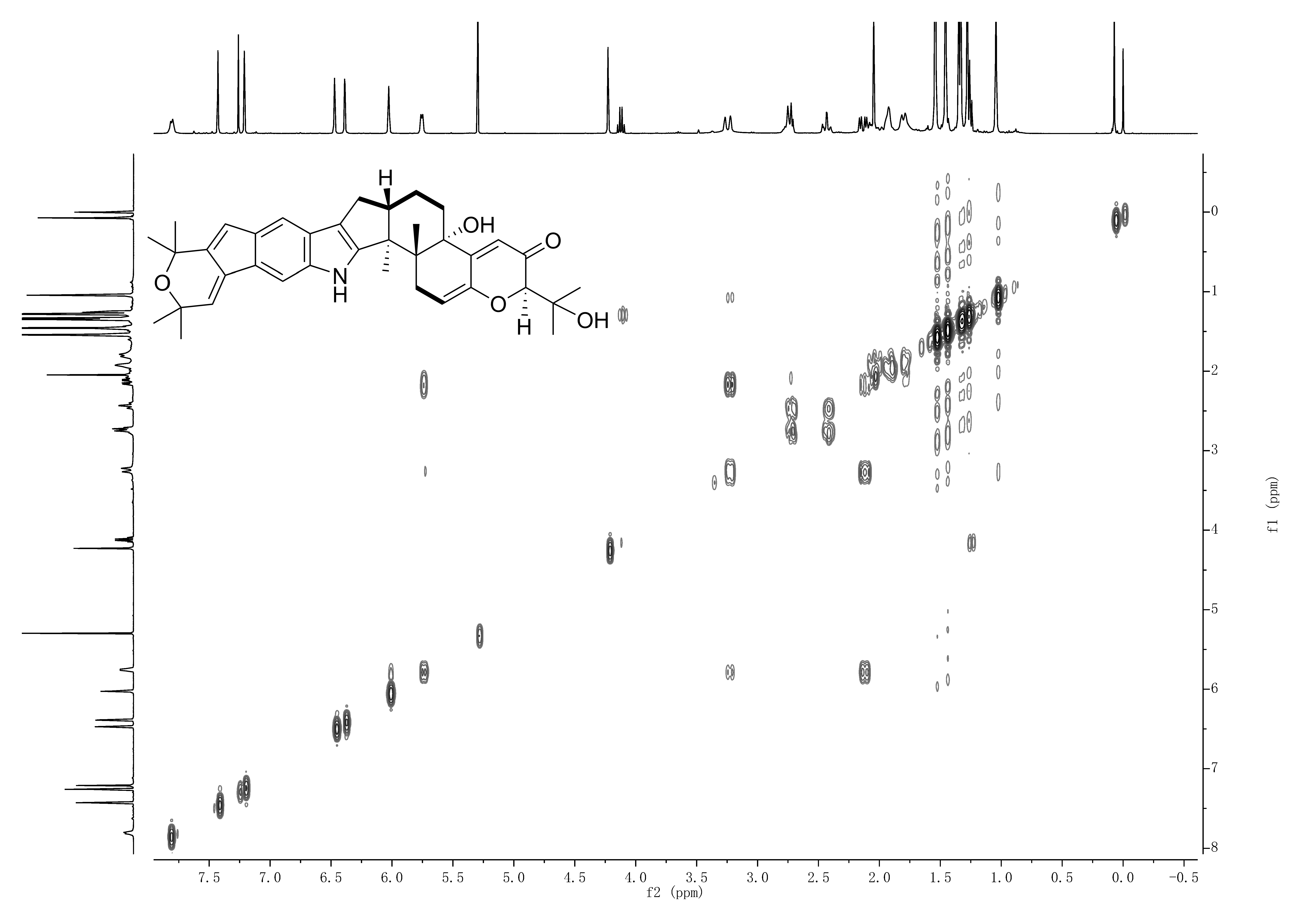


**Figure S4.** ^1^H–^1^H COSY (CDCl_3_) spectrum of compound **1**


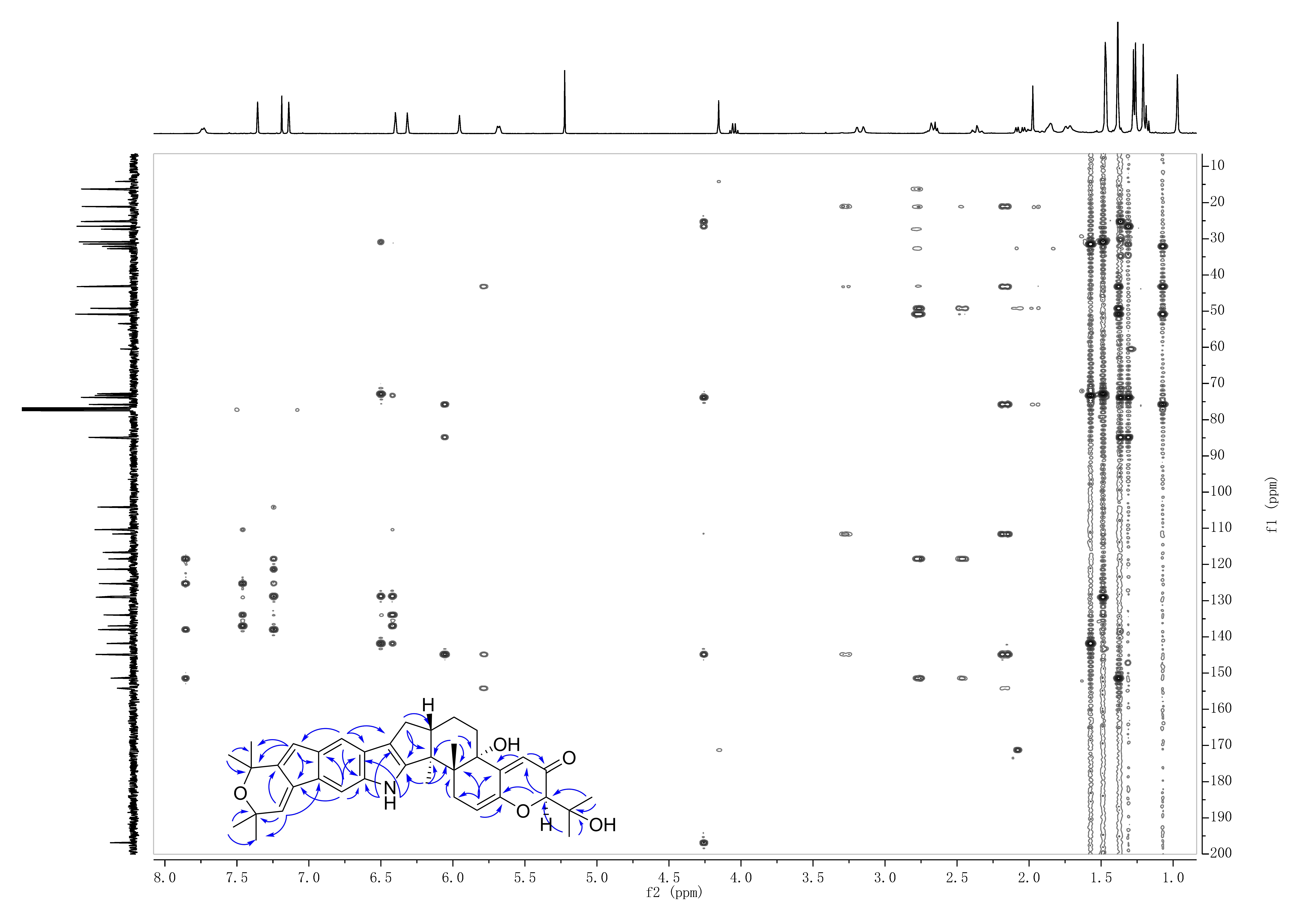


**Figure S5.** HMBC (CDCl_3_) spectrum of compound **1**


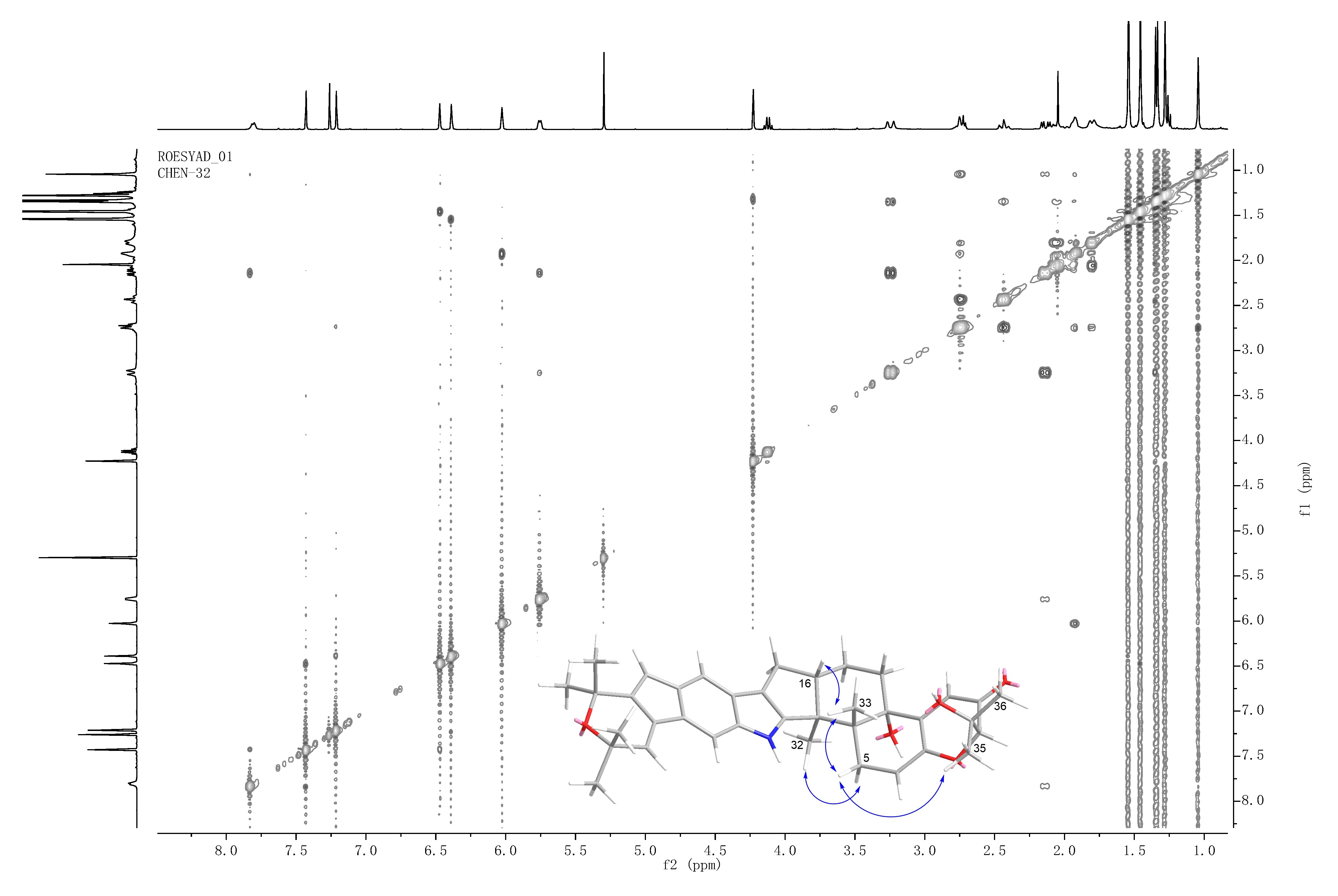


**Figure S6.** ROESY (CDCl_3_) spectrum of compound **1**

HRESIMS *m/z* 604.3042, [M + Na]^+^, (calcd for C_37_H_43_NNaO_5_^+^, 604.3033)

**Figure S7.** HRESIMS spectrum of compound **1**

**

**

**Figure S8**. ECD spectra of **1**


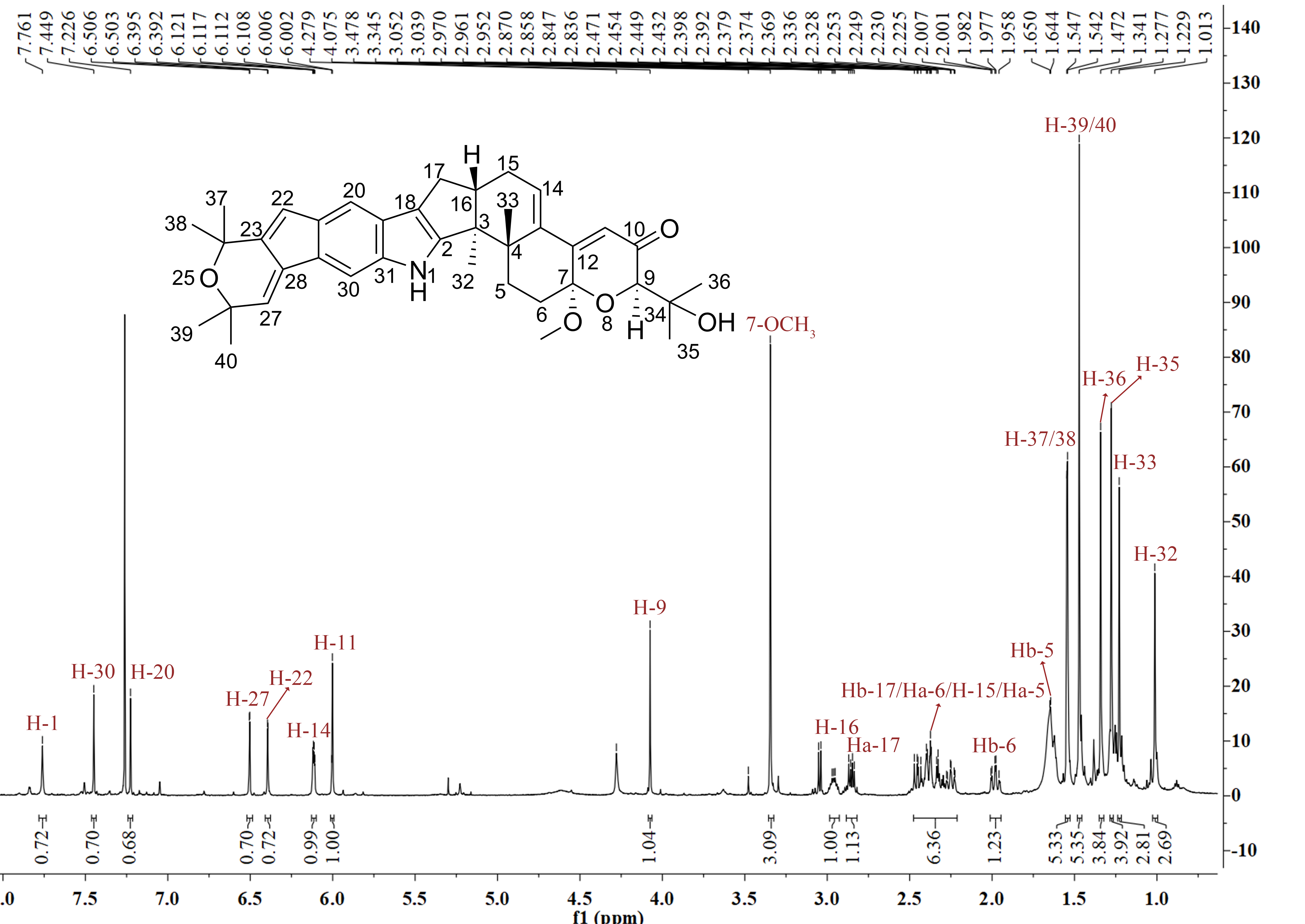


**Figure S9.** ^1^H NMR (600 MHz, CDCl_3_) spectrum of compound **2**


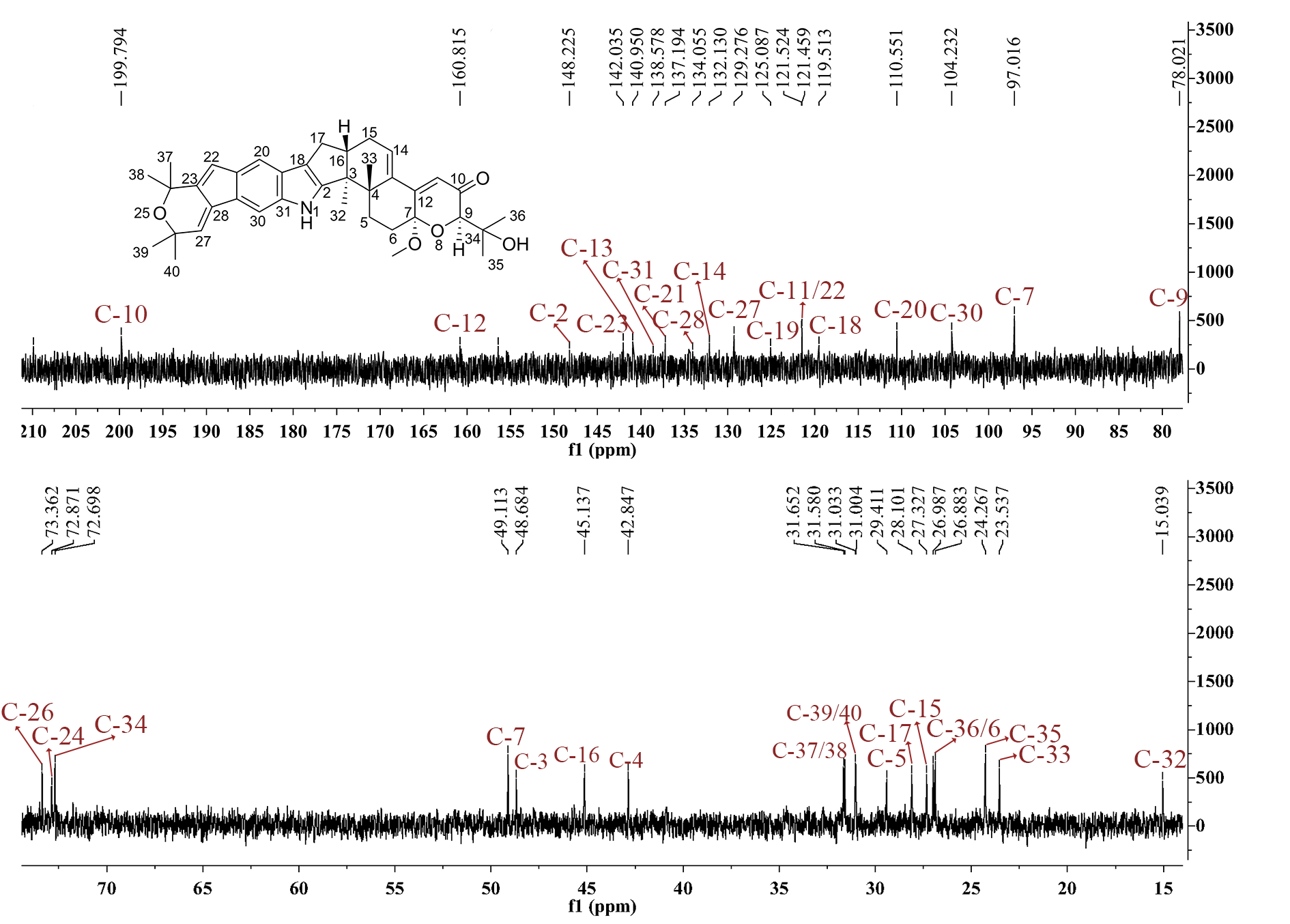


**Figure S10.** ^13^C NMR (150 MHz, CDCl_3_) spectrum of compound **2**


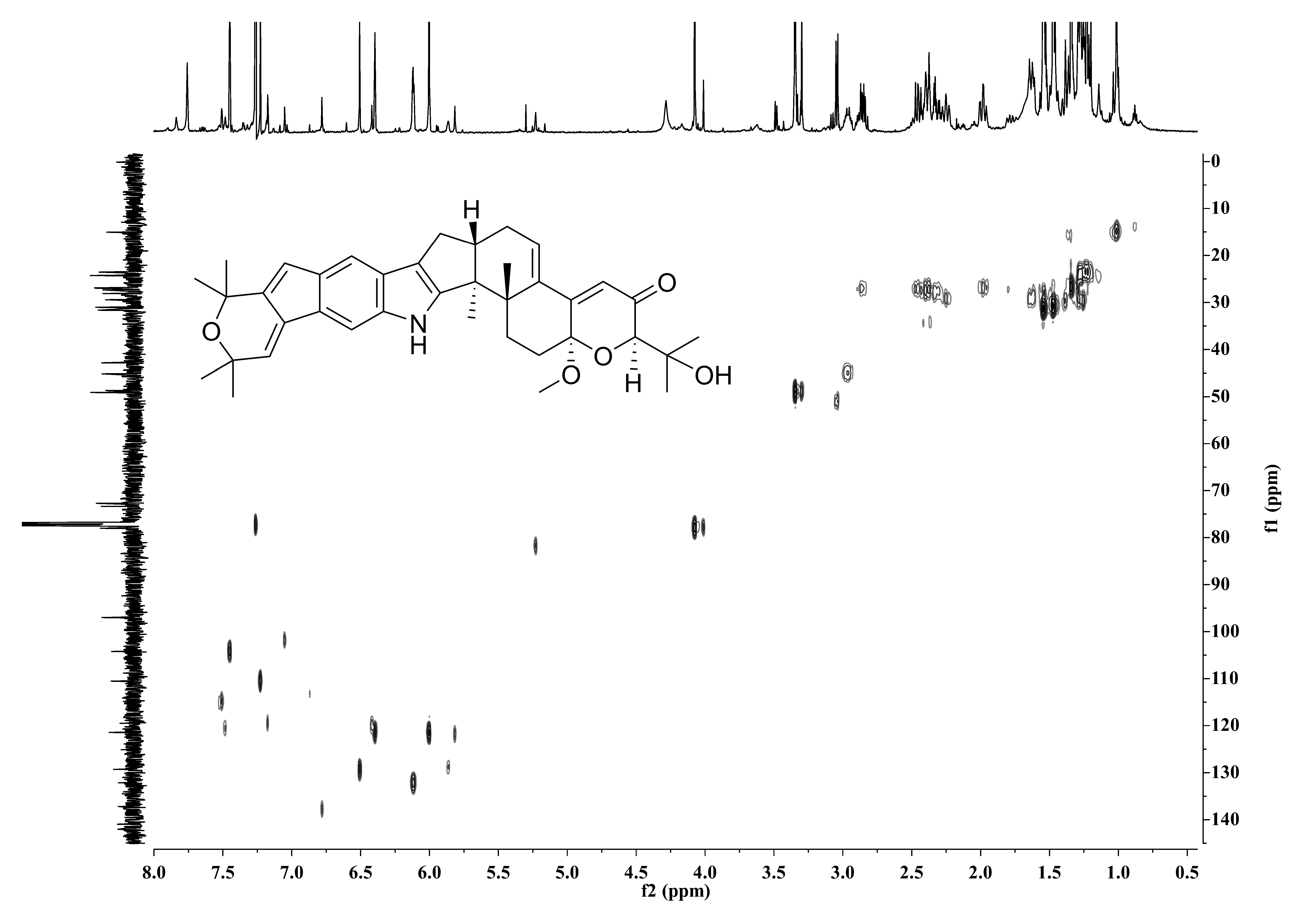


**Figure S11.** HSQC (CDCl_3_) spectrum of compound **2**


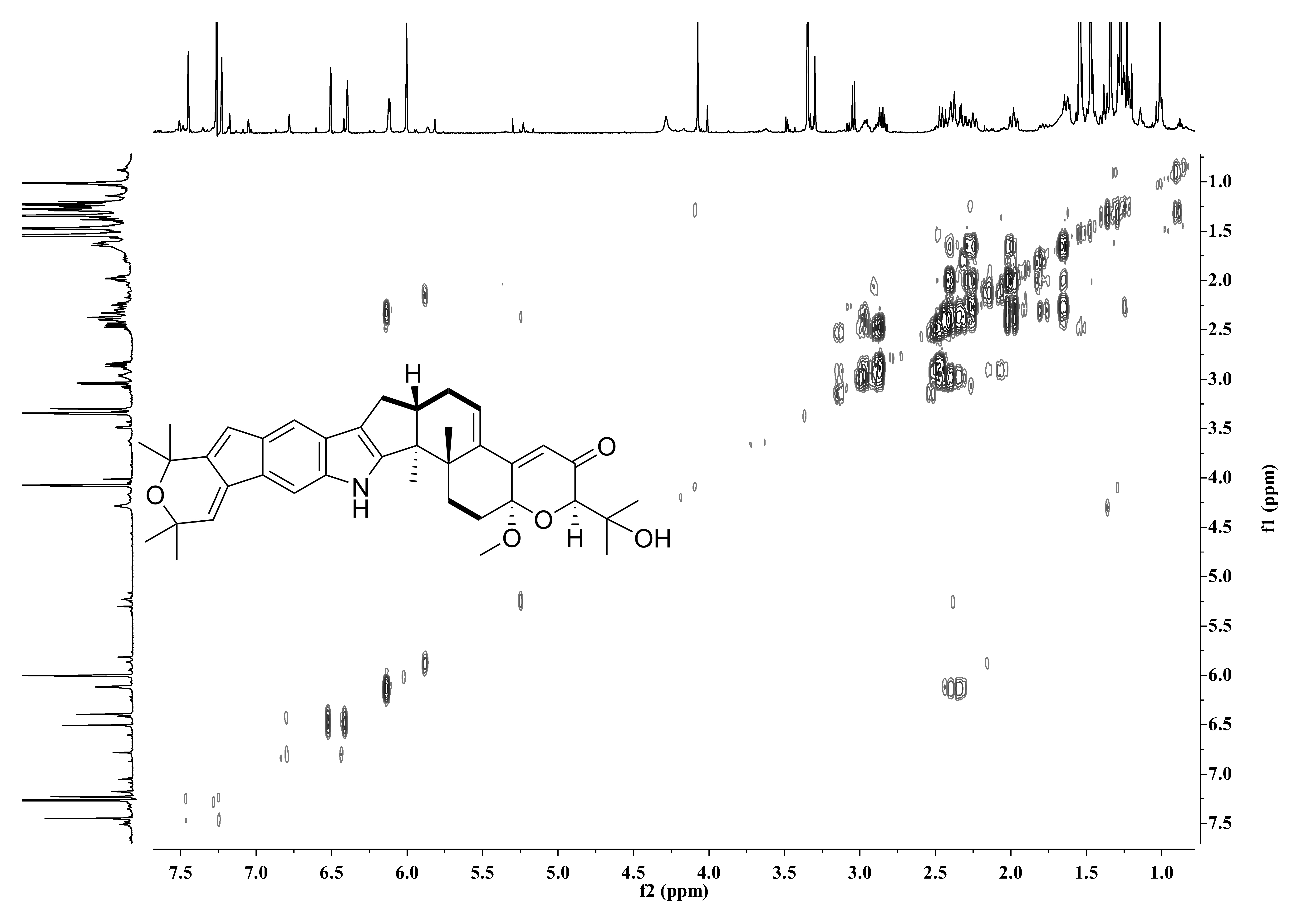


**Figure S12.** ^1^H–^1^H COSY (CDCl_3_) spectrum of compound **2**


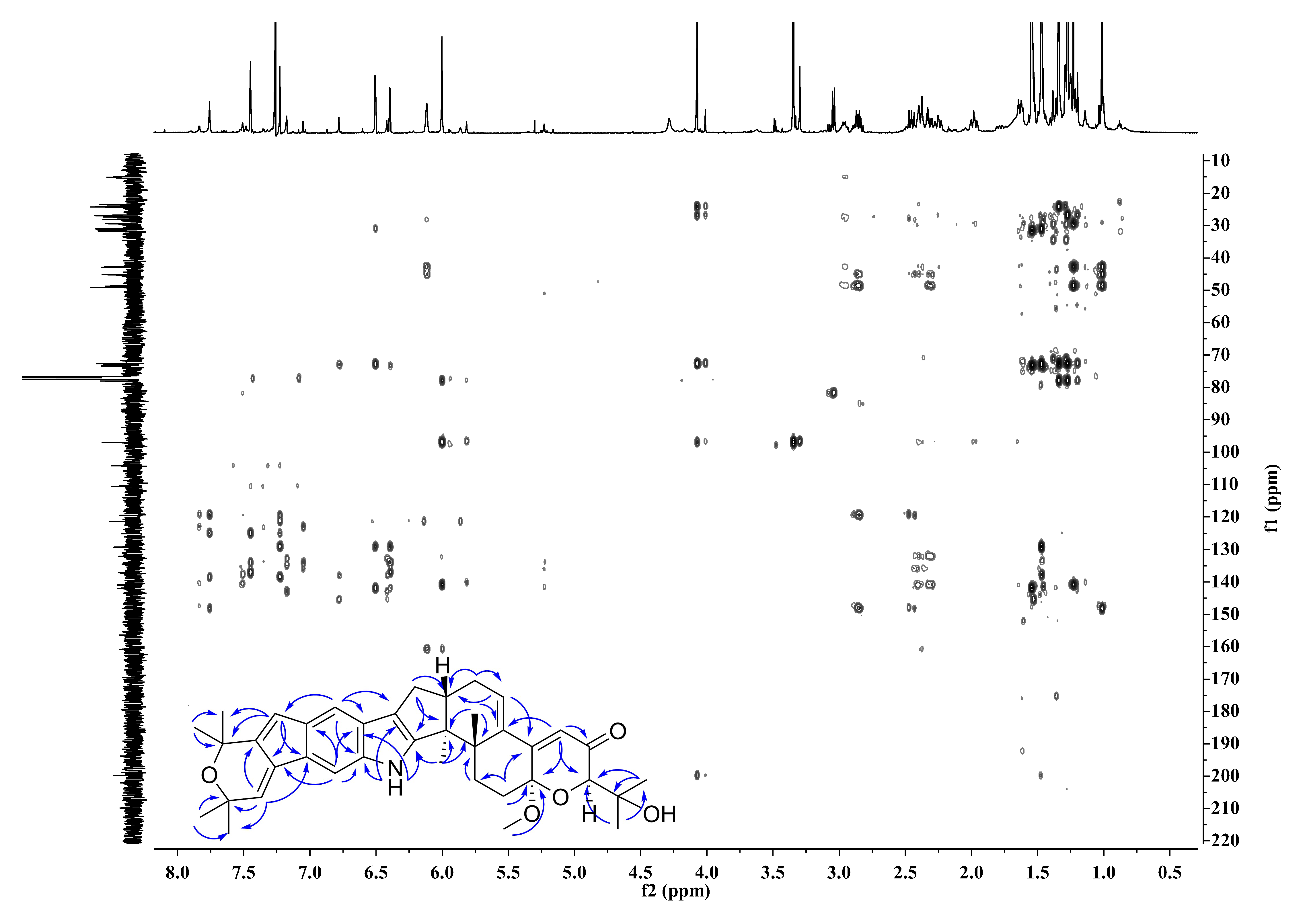


**Figure S13.** HMBC (CDCl_3_) spectrum of compound **2**


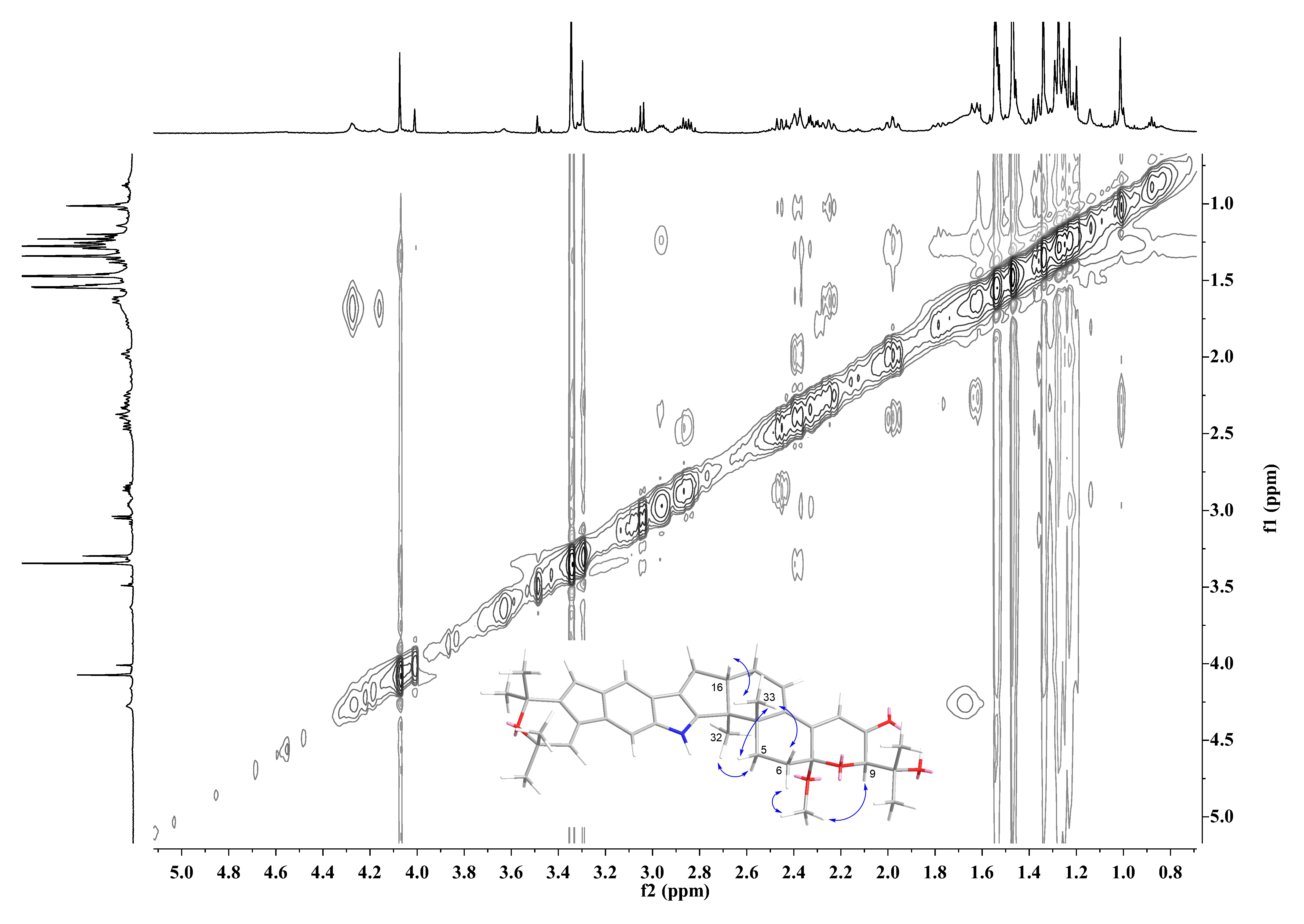


**Figure S14.** NOESY (CDCl_3_) spectrum of compound **2**

HRESIMS *m/z* 618.3187, [M + Na]^+^, (calcd for C_38_H_45_NNaO_5_^+^, 618.3190)

**Figure S15.** HRESIMS spectrum of compound **2**





**Figure S16**. ECD spectra of **2**


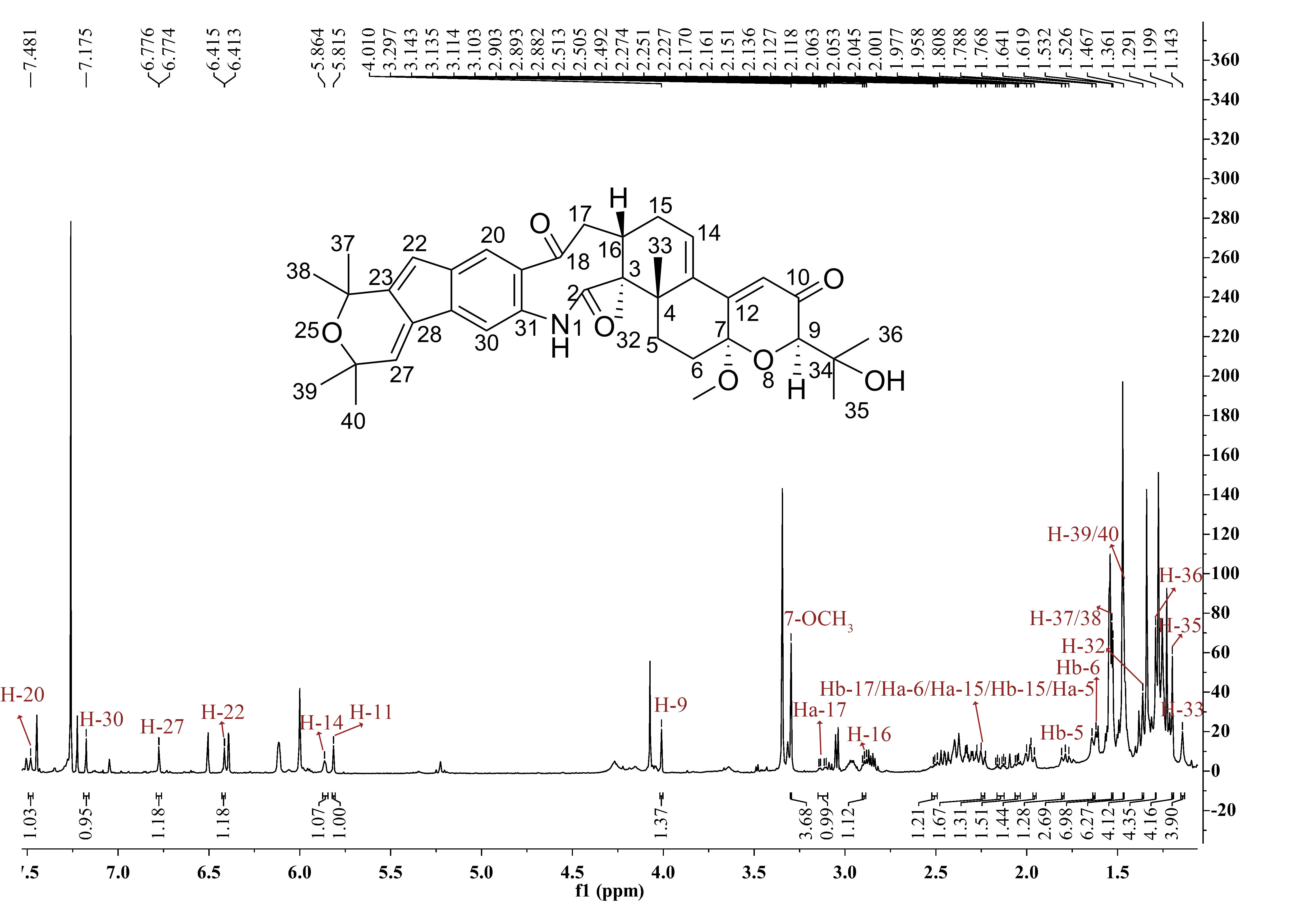


**Figure S17.** ^1^H NMR (600 MHz, CDCl_3_) spectrum of compound **3**

(A mixture of **3** and **2** (approx. 2:3, n:n), the unmarked signals belong to **2**)


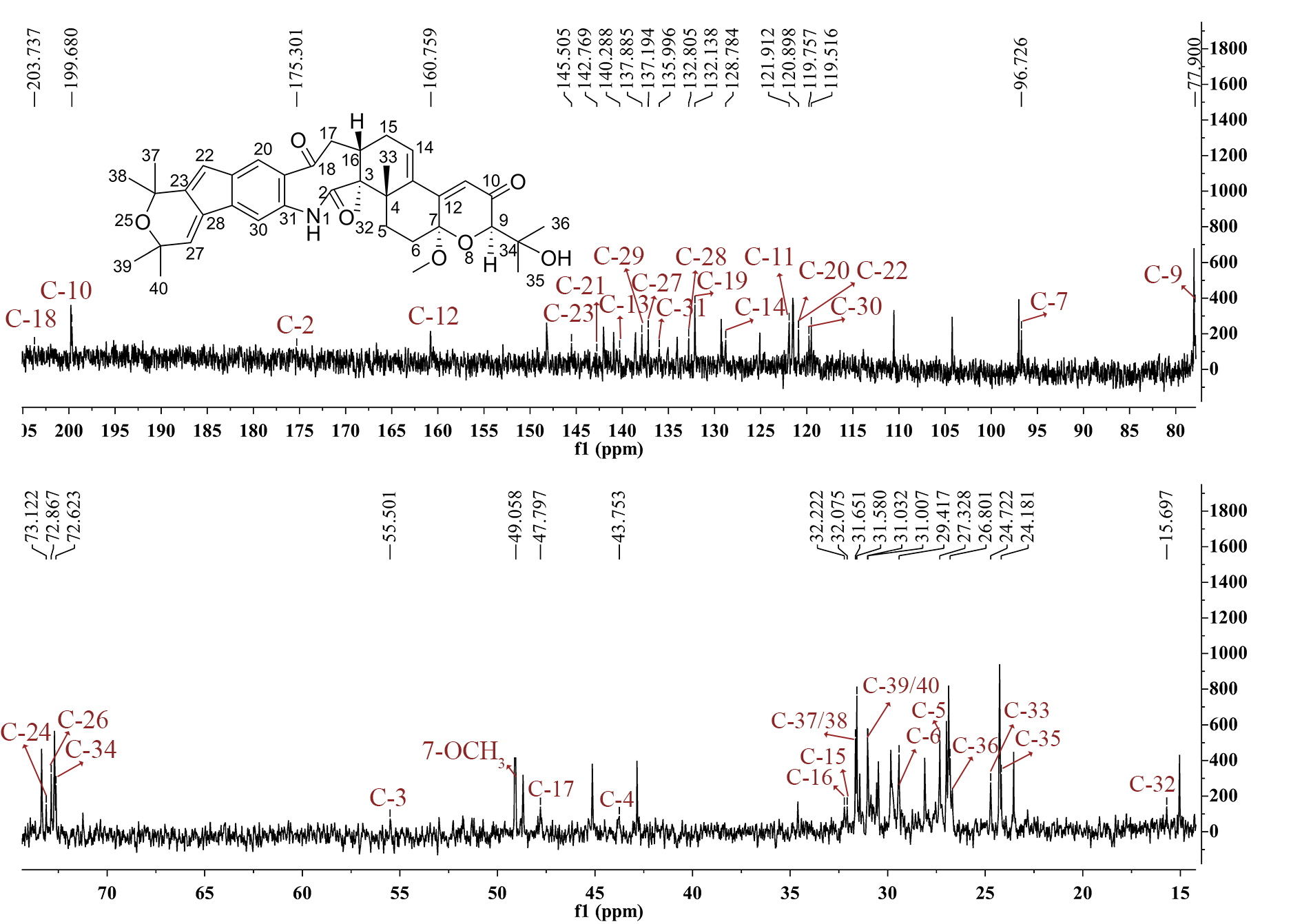


**Figure S18.** ^13^C NMR (150 MHz, CDCl_3_) spectrum of compound **3**

(A mixture of **3** and **2** (approx. 2:3, n:n), the unmarked signals belong to **2**)


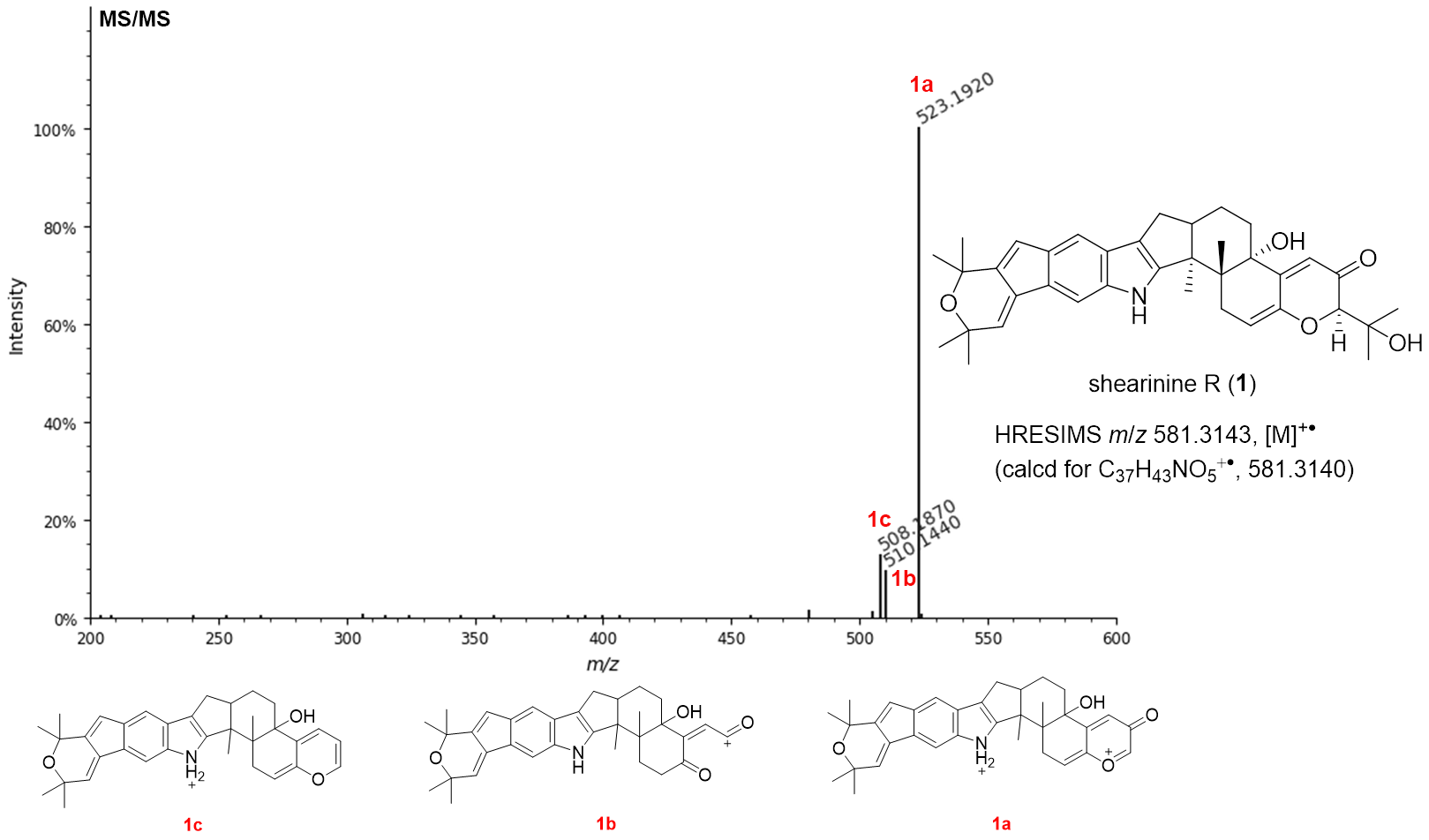


**Figure S19.** (Upper) MS/MS spectrum of shearinine R (**1**); (Under) MS/MS fragment ions of shearinine R (**1**)

**
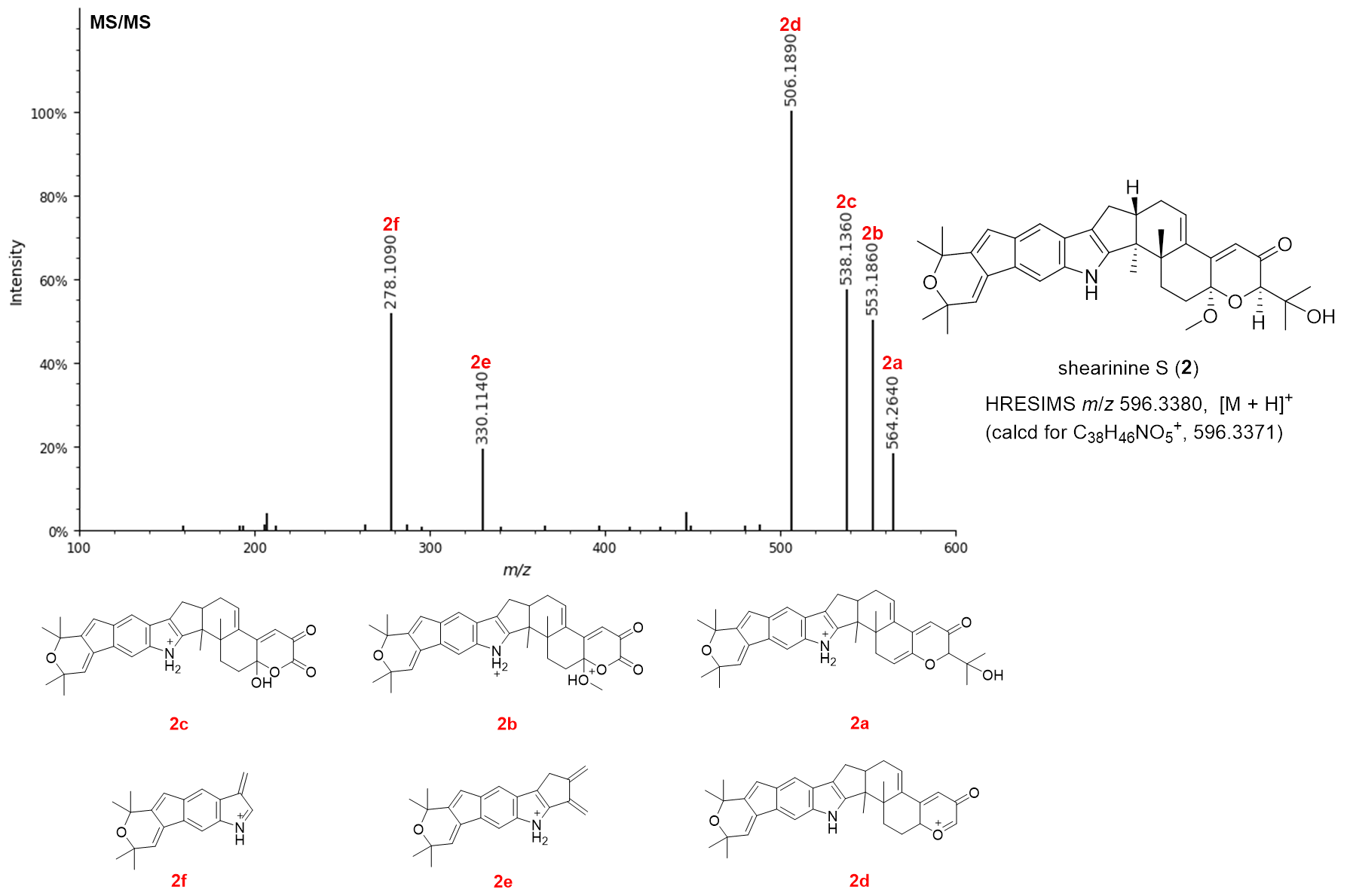
**

**Figure S20.** (Upper) MS/MS spectrum of shearinine S (**2**); (Under) MS/MS fragment ions of shearinine S (**2**)

**
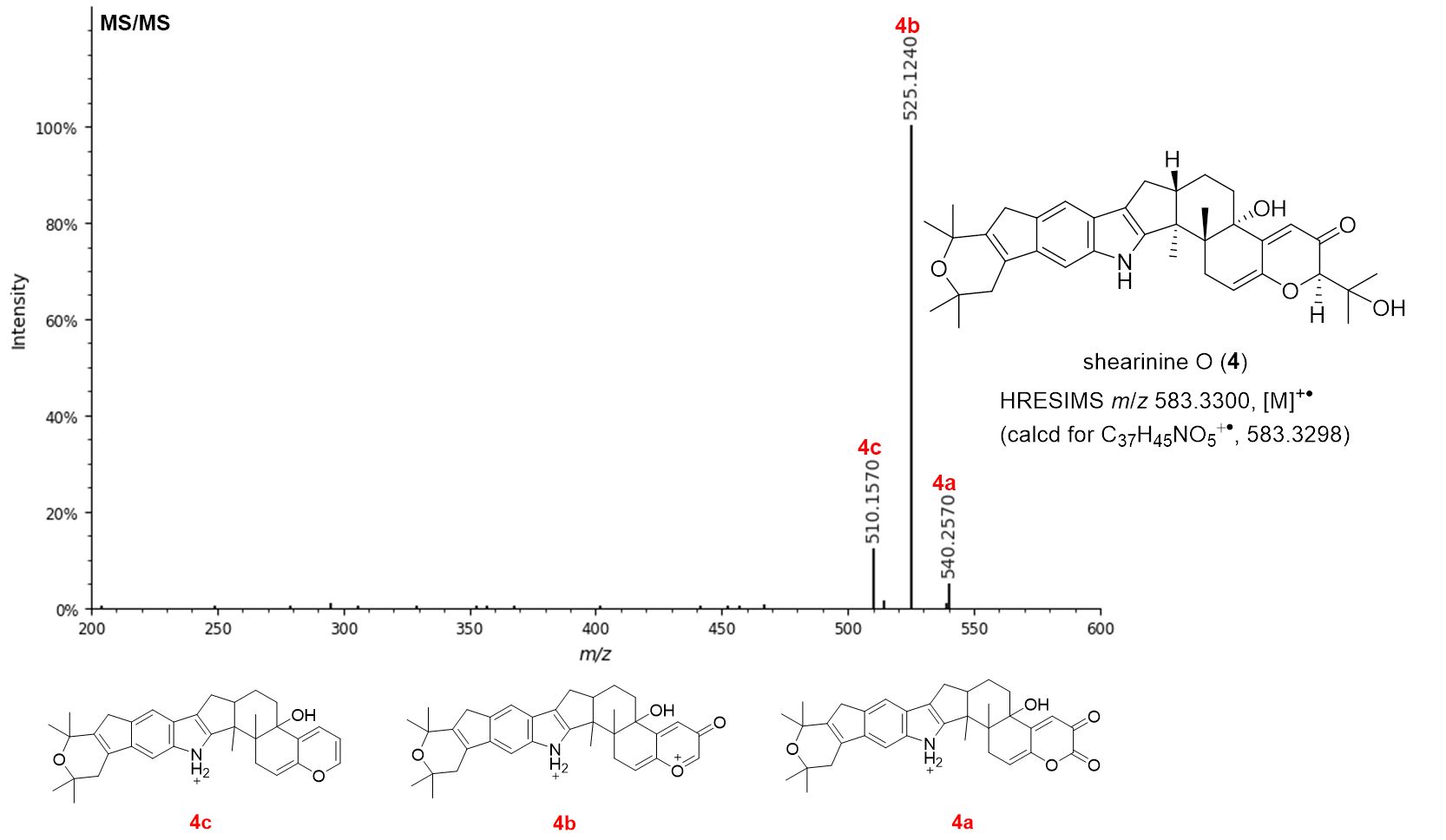
**

**Figure S21.** (Upper) MS/MS spectrum of shearinine O (**4**); (Under) MS/MS fragment ions of shearinine O (**4**)


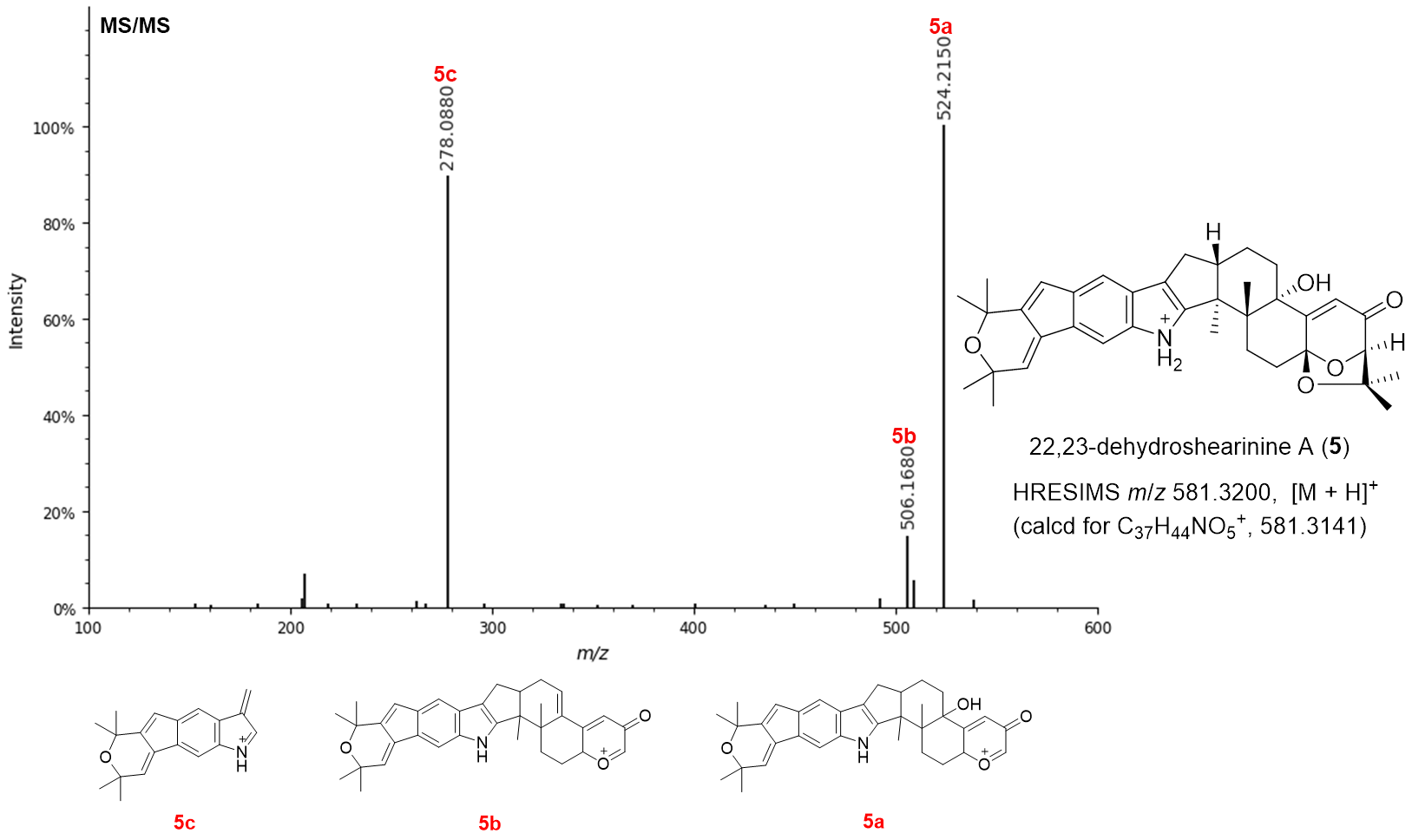


**Figure S22.** (Upper) MS/MS spectrum of 22,23-dehydroshearinine A (**5**); (Under) MS/MS fragment ions of 22,23-dehydroshearinine A (**5**)

**
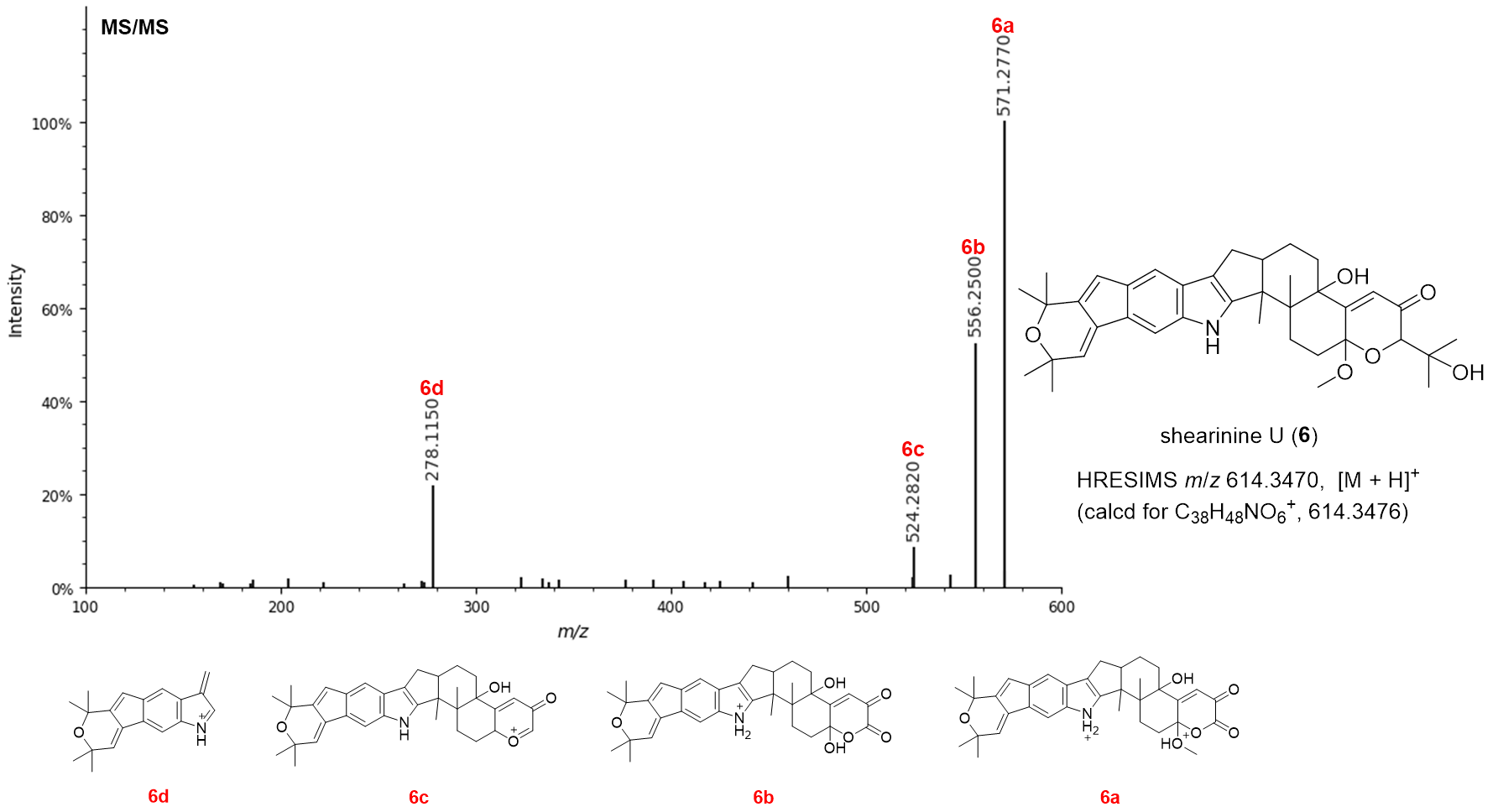
**

**Figure S23.** (Upper) MS/MS spectrum of shearinine U (**6**); (Under) MS/MS fragment ions of shearinine U (**6**)

**
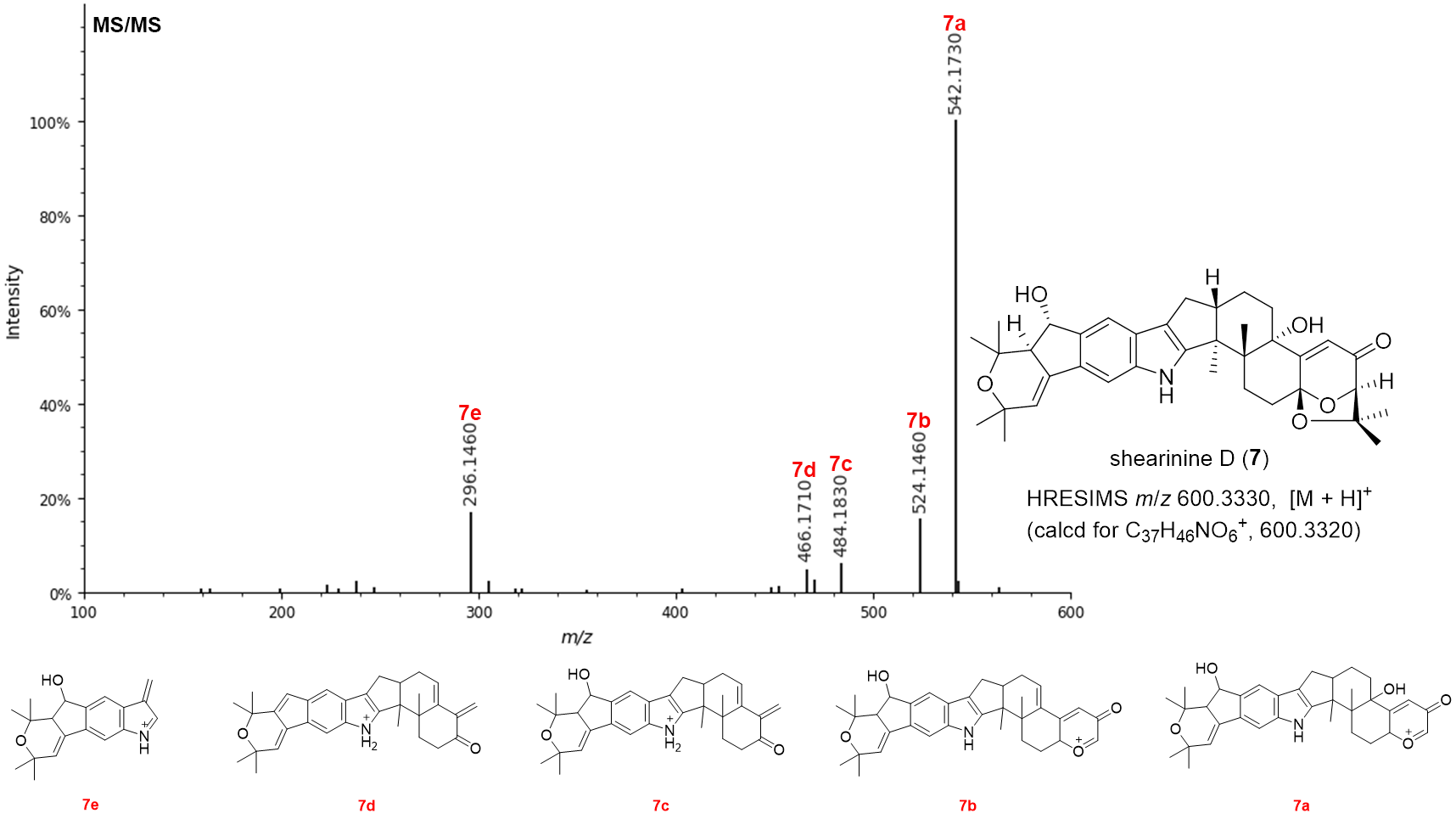
**

**Figure S24.** (Upper) MS/MS spectrum of shearinine D (**7**); (Under) MS/MS fragment ions of shearinine D (**7**)

**
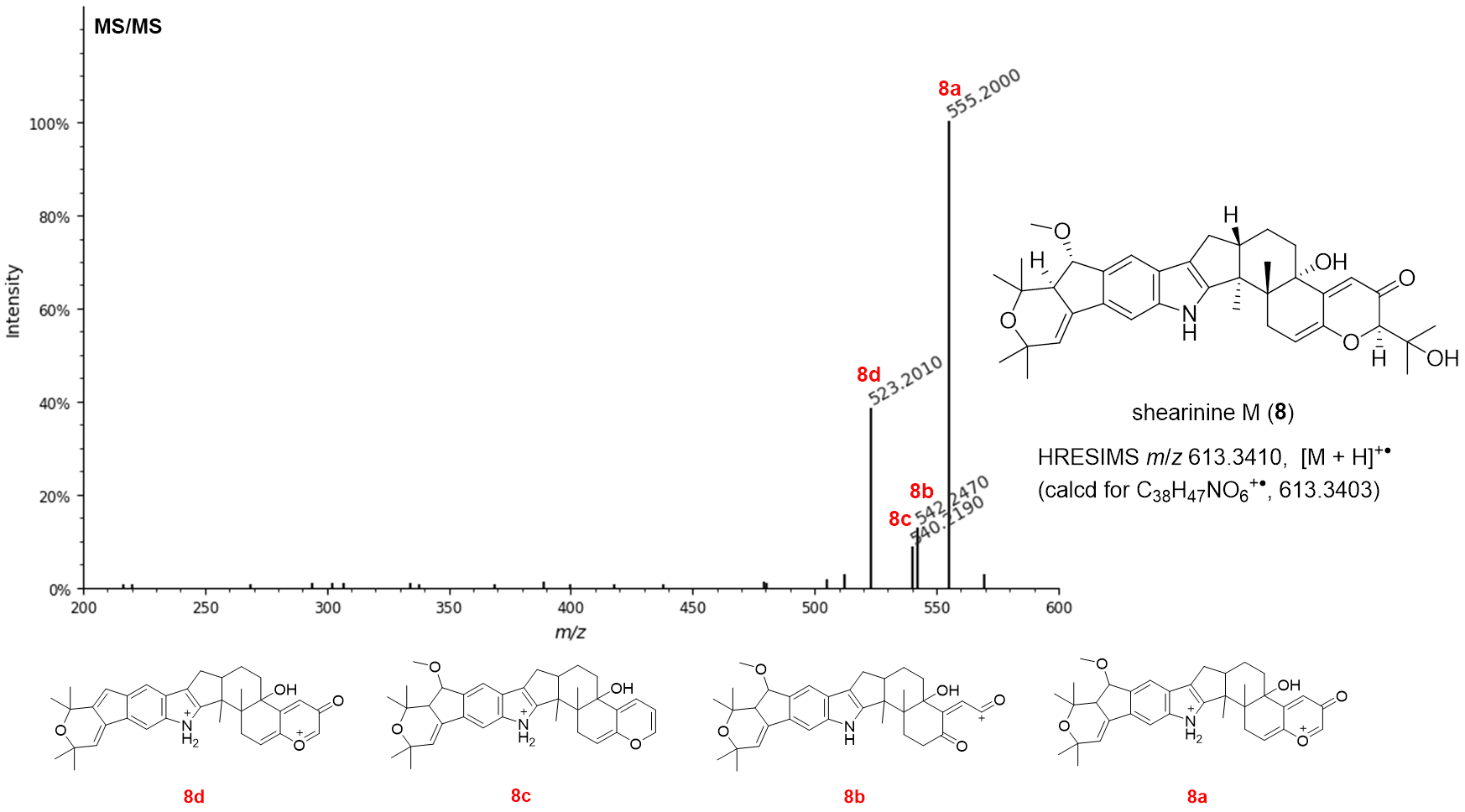
**

**Figure S25.** (Upper) MS/MS spectrum of shearinine M (**8**); (Under) MS/MS fragment ions of shearinine M (**8**)


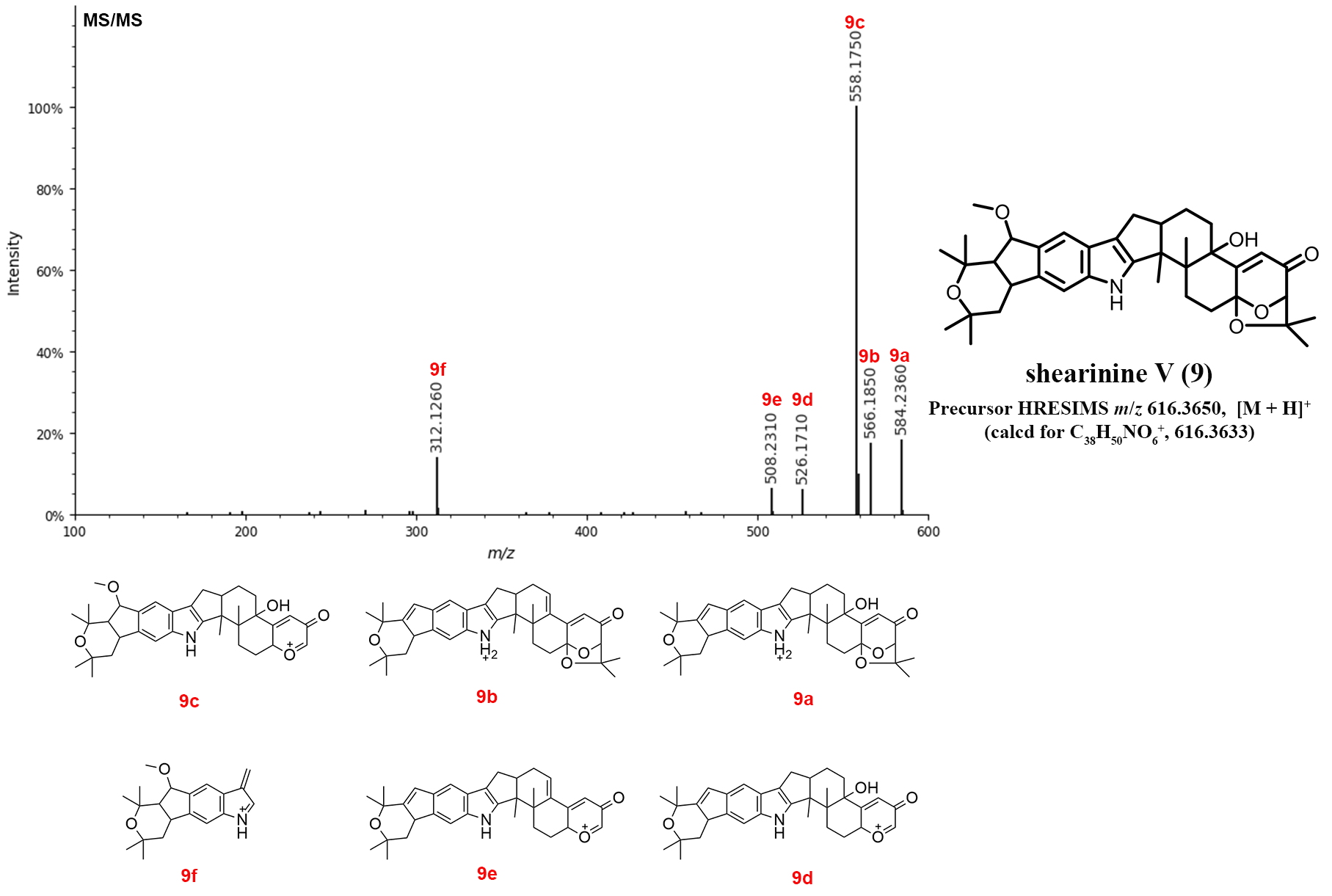


**Figure S26.** (Upper) MS/MS spectrum of shearinine V (**9**); (Under) MS/MS fragment ions of shearinine V (**9**)

**
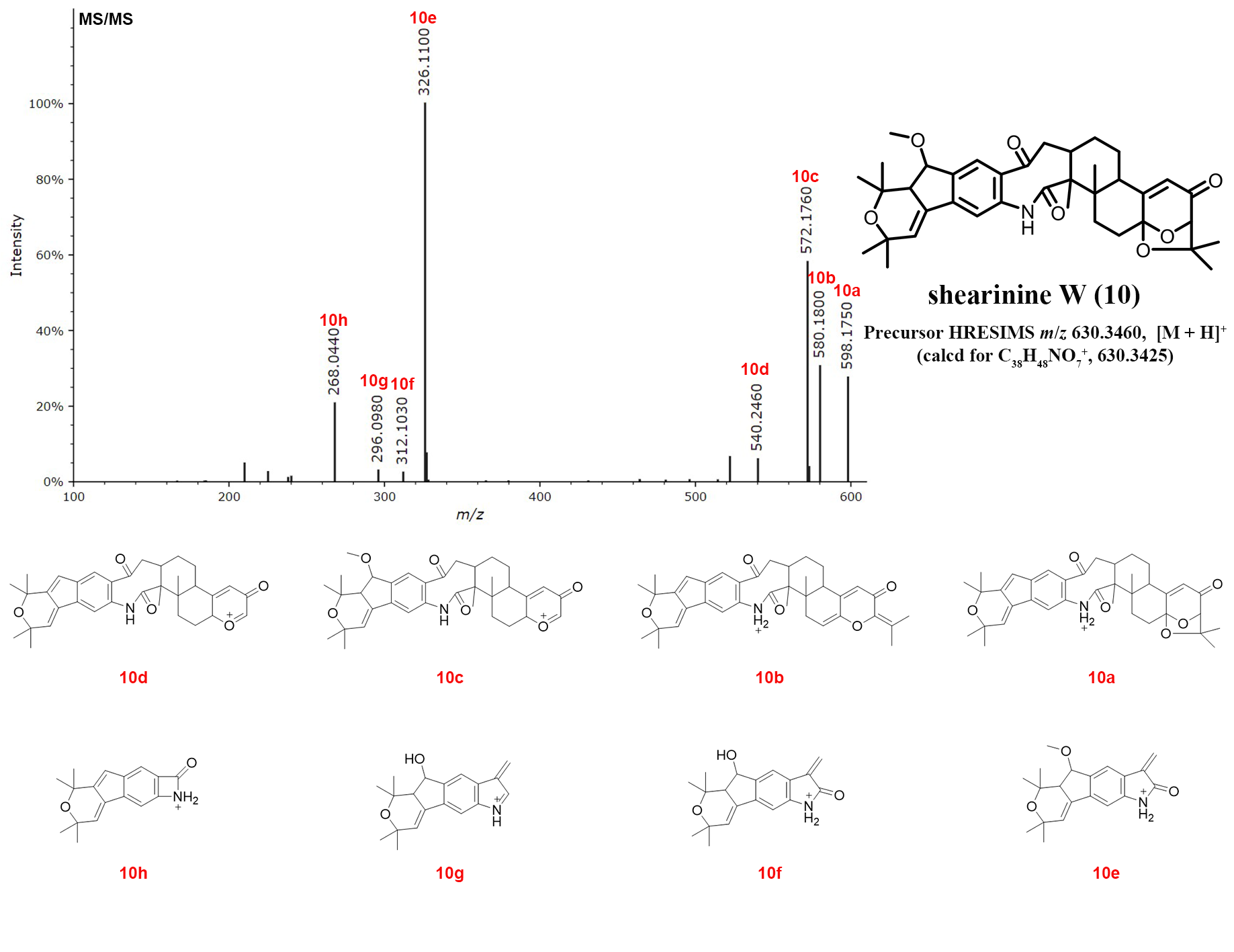
**

**Figure S27.** (Upper) MS/MS spectrum of shearinine W (**10**); (Under) MS/MS fragment ions of shearinine W (**10**)


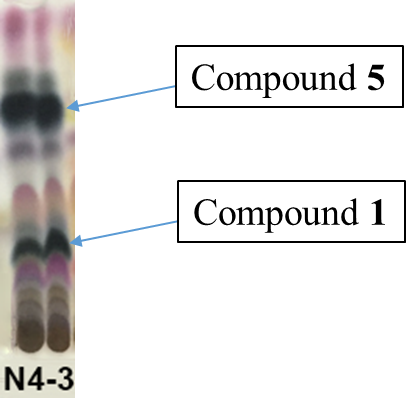


**Figure S28.** The thin layer chromatography analysis of the fungal extracts from *Penicillium* sp. N4-3

(Compounds **1** and **5** are the major products.)

**
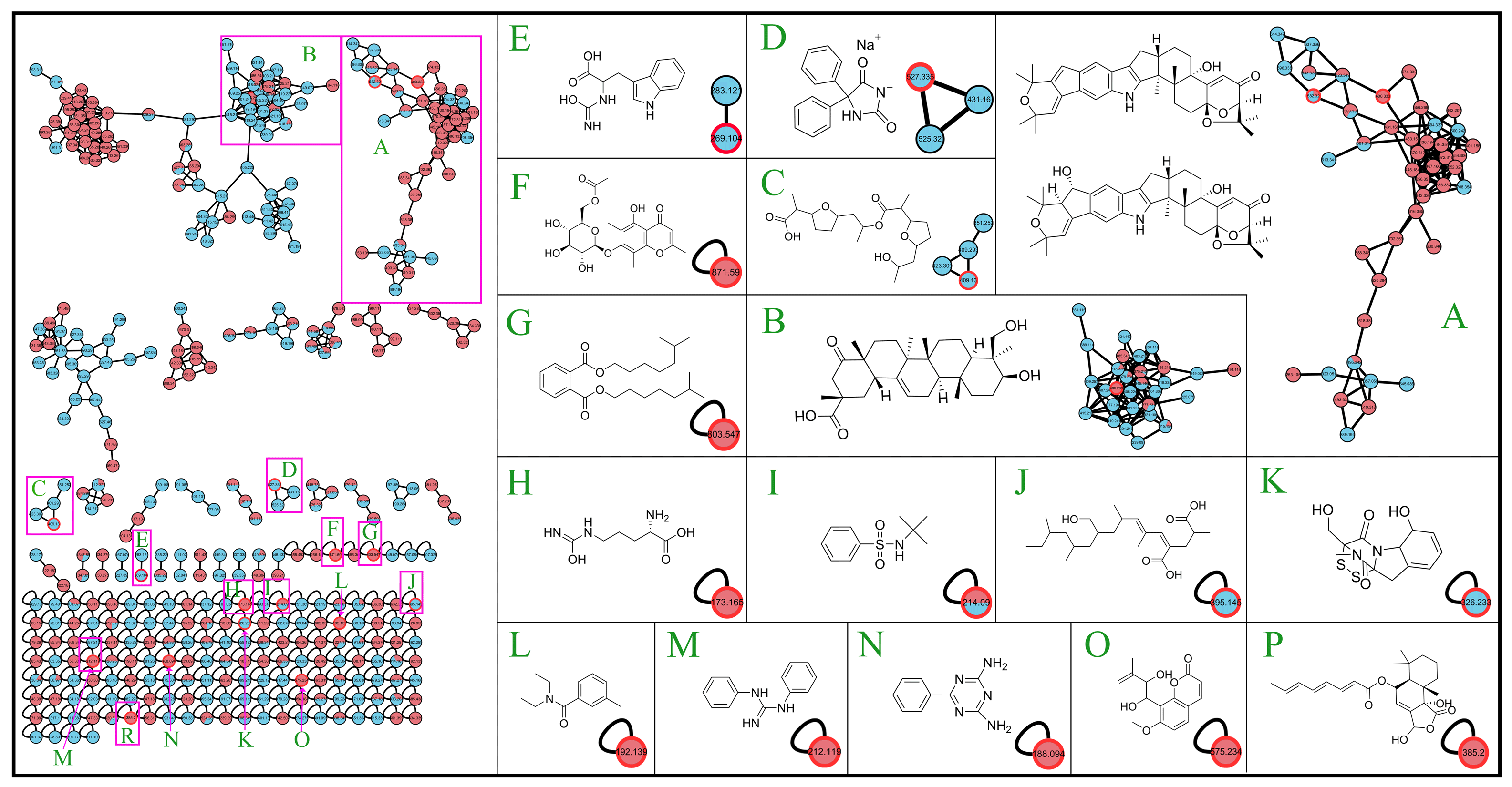
**

**Figure S29.** Full molecular networking of the profiled compounds from two fractions of the fungal extracts from *Penicillium* sp. N4-3 annotated by GNPS database (red nodes from Fr. 1, and blue nodes from Fr. 2)

*Penicillium simplicissimum* isolate 79 (MH137657.1)

*Penicillium janthinellum* strain sm-12f4 (GU212865.1)

N4-3

*Penicillium cf. piscarium* DTO 108-E1 18S (KC346343.1)

*Penicillium ochrochloron* isolate 153 (MH137683.1)

*Penicillium rolfsii* strain SFCF20120912-04 (KF313082.1)

*Penicillium levitum* isolate dmAE (MF567516.1)

*Paecilomyces victoriae* culture CBS:469.70 (MH859799.1)

*Penicillium pedernalense* isolate WZ-919 (OP163778.1)

*Penicillium expansum* isolate BK225 (KU702681.1)

*Penicillium pulvillorum* strain CMV007G4 (MK450709.1),

94

93

59

88

45

46

63

0.001

**Figure S30.** Phylogenetic tree of ITS rRNA sequences of closely related *Penicillium* sp. N4-3. Reference sequences were downloaded from NCBI with the accession numbers indicated in parentheses. Distances and clustering was performed by MEGA 10. Bootstrap values based on 1000 replications are listed at the branching point.
